# High H_2_ Solubility of Perfluorocarbon Solvents and Their Use in Reversible Polarization Transfer from *para*-Hydrogen

**DOI:** 10.1021/acs.jpclett.4c03190

**Published:** 2025-01-06

**Authors:** Callum A. Gater, Orry J. Mayne, Benjamin G. Collins, Kieren J. Evans, Eleanor M. E. Storr, Adrian C. Whitwood, Daniel P. Watts, Ben J. Tickner, Simon B. Duckett

**Affiliations:** † Centre for Hyperpolarization in Magnetic Resonance, University of York, Heslington YO10 5NY, United Kingdom; ‡ Department of Chemistry, University of York, Heslington YO10 5DD, United Kingdom; § Department of Physics, Engineering and Technology, University of York, Heslington YO10 5DD, United Kingdom

## Abstract

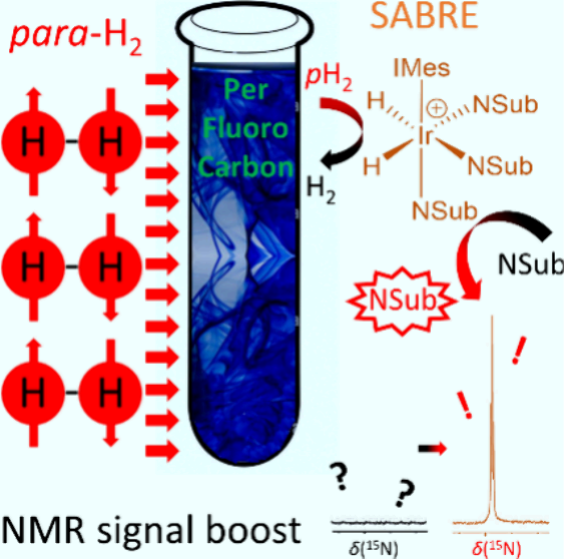

This research uses perfluorocarbons (PFCs) as effective alternatives to traditional toxic solvents in reversible *para*-hydrogen-induced polarization (PHIP) for NMR signal enhancement. Hydrogen solubility in PFCs is shown here to be an order of magnitude higher than in typical organic solvents by determination of Henry’s constants. We demonstrate how this high H_2_ solubility enables the PFCs to deliver substantial polarization transfer from *para*-hydrogen, achieving up to 2400-fold signal gains for ^1^H NMR detection and 67,000-fold (22% polarization) for ^15^N NMR detection at 9.4 T in substrates such as pyridine and nicotine. Notably, methylperfluorobutylether outperforms catalytic efficiency in methanol-*d*_4_ and dichloromethane-*d*_2_ for pyridine at low catalyst loadings. This makes PFCs particularly advantageous for applications demanding high NMR sensitivity. With high polarization efficiency and reduced toxicity, PFCs hold strong potential for expanding hyperpolarized NMR applications across the biomedical and analytical fields.

Magnetic resonance (MR) methods are used widely across the physical sciences to investigate molecular structure and dynamics due to the high information content of the data they provide.^1^ However, they suffer from an intrinsic sensitivity limitation as these methods interrogate nuclear spin orientations that lie close in energy, and hence concentrated samples and/or signal averaging is typically required for analysis.^2^ One approach to improve MR sensitivity is to create transient non-Boltzmann spin-energy level populations, often termed “hyper”-polarization, with the result that detected MR signals become significantly larger than those normally recorded.^2^ For example, only one in every *ca* 300,000 ^15^N nuclei is effectively detected using thermally polarized NMR at 9.4 T due to the minuscule population difference defining the associated energy levels. However, hyperpolarization methods delivering 50% polarization or higher, which reflect a sensitivity improvement of 150,000-fold for this nucleus, have been reported.^3−7^ A clear advantage of this approach is that by transiently boosting the NMR signal intensity, molecules with short lifetimes (seconds) or low concentrations (down to picomolar) can be detected in single-scan MR experiments. This benefit provides significant opportunities for mechanistic elucidation, intermediate detection, catalysis, reaction monitoring, chemosensing, and even biomedical imaging.^8−11^

Here, we develop the hyperpolarization method signal amplification by reversible exchange (SABRE) which exploits reversible interactions between a metal catalyst and both *para*-hydrogen (*p*H_2_) and a to-be-hyperpolarized molecule.^12−14^ This process unleashes the latent magnetism that is locked within *p*H_2_ to produce a hyperpolarized metal dihydride. Its *J* coupling network, that exists between the hydride ligands and the spins of a ligand, can result in ligand hyperpolarization, which survives its dissociation into solution (Figure 1a). As the process of SABRE is catalytic, multiple polarization events can accumulate significant polarization levels for proton,^15,16^ nitrogen,^3−7^ and carbon^17^ (and to a lesser extent fluorine,^18−20^ silicon,^21,22^ phosphorus,^23,24^ and tin^22^) nuclei in seconds. These are reflected in the fact a range of molecular types, including *N*-heterocycles, amines, diazirines, nitriles, imines, and ketoacids have proven suitable as targets.^14,25^

**Figure 1:**
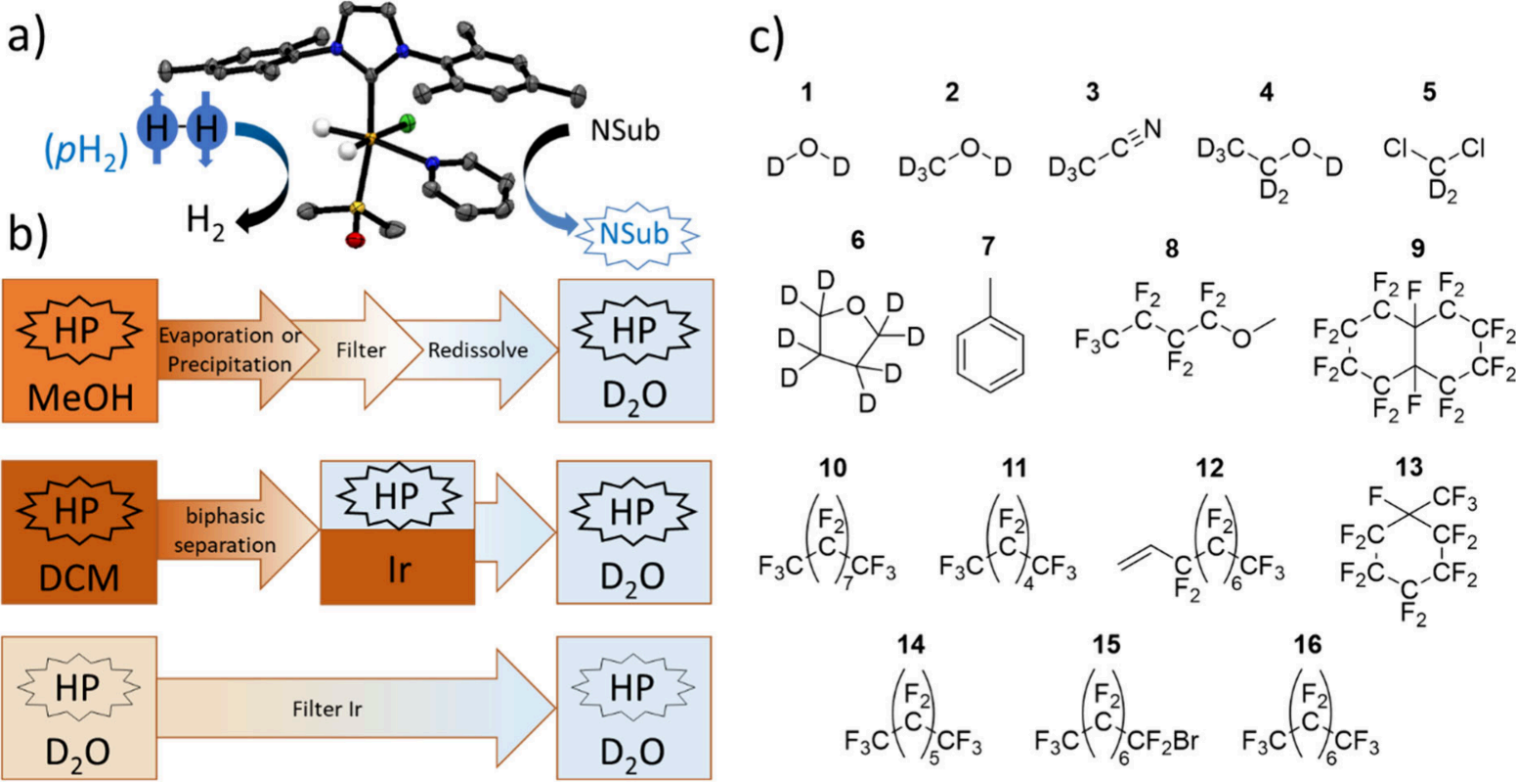
(a) Depiction of the SABRE process, which boosts the NMR signals of target molecules if they and *para*-hydrogen are in reversible exchange with a catalyst where NSub is an N-heterocycle, e.g., pyridine. In this example, the X-ray crystal structure of the active SABRE catalyst [IrCl(H)_2_(DMSO)(IMes)(pyridine)] is shown. (b) Summary of the current methods used to produce a biocompatible bolus suitable for *in vivo* biomedical imaging. These include (upper) hyperpolarization in a methanolic solvent followed by sample concentration via solvent evaporation, filtration of the precipitated catalyst, and redissolution to generate a catalyst-free aqueous bolus.^26^ Instead, the hyperpolarized agent could be precipitated and filtered out of the solution containing catalyst before being redissolved.^27^ Alternatively, (middle) hyperpolarization in a chlorinated solvent followed by biphasic separation which retains the catalyst in the chlorinated solvent and allows the hyperpolarized agent to move into the aqueous phase where it is collected^28^ and (lower) hyperpolarization directly in aqueous solvent using a water-soluble SABRE catalyst which can be filtered out.^31,32^ (c) Solvents examined in this work.

SABRE is typically performed in methanol-*d*_4_, dichloromethane-*d*_2_, or chloroform-*d* solvents as they allow both the iridium SABRE catalyst and *p*H_2_ to be solubilized. However, such solvents present a challenge to those seeking to create hyperpolarized boluses for biomedical applications, as they are toxic. Most solutions to this issue have sought to perform the hyperpolarization step in an organic solvent, prior to solvent evaporation/precipitation/redissolution^26,27^ or phase separation/extraction^28^ to create the necessary biocompatible aqueous solution (Figure 1b). While this can be successful, there are challenges associated with polarization losses during the preparation period where *T*_1_ relaxation operates and H_2_ concentration in solution can often limit the efficiency of SABRE catalysis.^16,29,30^

While hyperpolarization in D_2_O might therefore be advantageous, direct hyperpolarization in this solvent using water-soluble catalysts has so far only produced a fraction of the MR signal enhancement achievable in these organic solvents with SABRE efficiency likely hampered by lower *p*H_2_ solubility.^31,32^ Other approaches have used organic solvents for catalyst activation, before the active SABRE species is collected by solvent evaporation and redissolved in D_2_O where the active Ir species is slightly more soluble than its precursor.^33^ However, a lower H_2_ solubility in D_2_O is still a factor that must be overcome.

There is therefore a clear benefit to developing highly H_2_-solubilizing solvents that also exhibit high catalyst solubility, long nuclear spin relaxation for dissolved molecules, and biocompatibility. The candidates selected are perfluorocarbons (**8**–**16**, Figure 1c) as they are known to act as oxygen carriers^34^ and exhibit low toxicity,^35^ with existing uses in medical applications such as blood substitutes during surgery.^34,36^ Some perfluorocarbons are known to act as good H_2_ carriers.^37,38^ The hydrogen solubilities and the efficiency of SABRE catalysis are assessed for the test substrate pyridine in this series of perfluorocarbons (**8**–**16**) and compared to commonly used organic solvents (**1**–**7**). The dissolved H_2_ gas concentration and corresponding mole fraction for a series of NMR tubes containing these solvents with varying pressures of H_2_ was determined using ^1^H NMR by comparison to an internal standard of known concentration (Figure 2). Henry’s law constants (*H*) (Table 1) were then calculated based on the linear relationship between gas vapor pressure (*p*) and measured concentration (*c*) according to eq 1.^39−46^
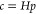
1

**Figure 2:**
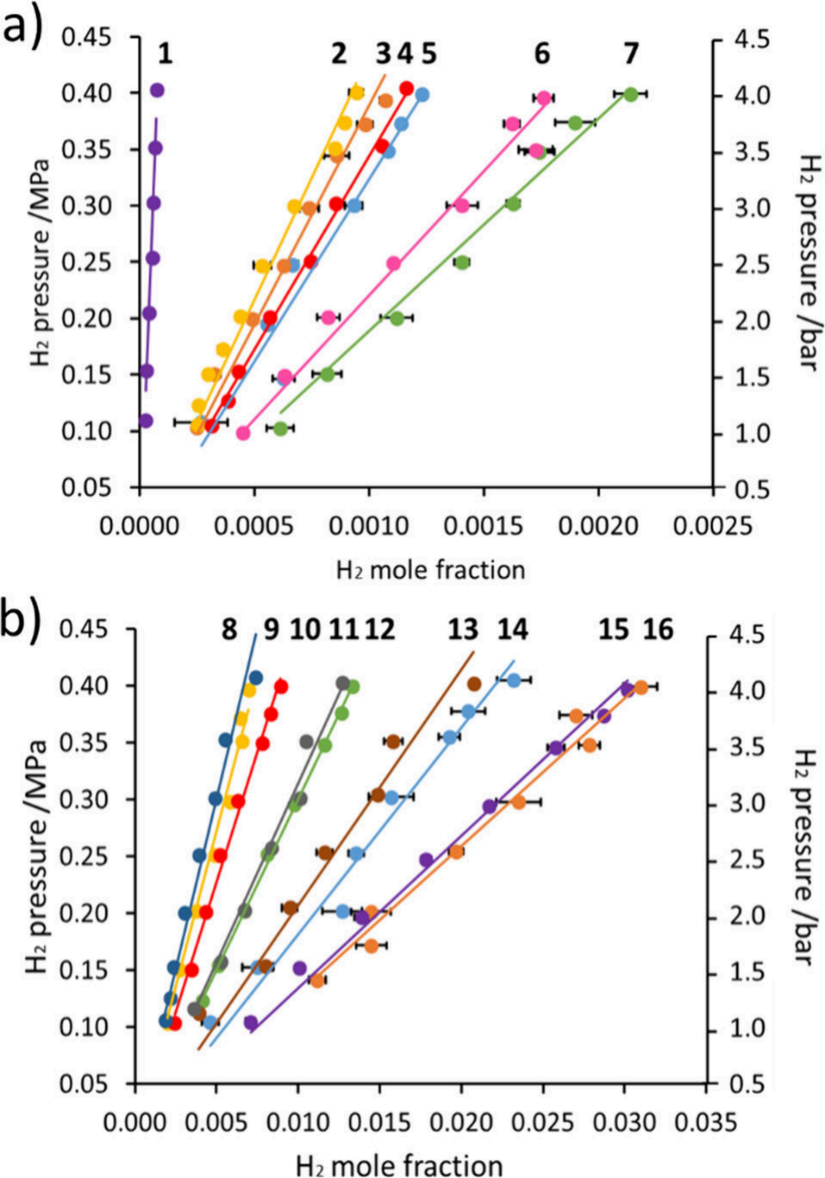
H_2_ mole fraction as a function of H_2_ pressure when dissolved in (a) organic solvents **1**–**7** and (b) perfluorocarbons **8**–**16**. The structures of these solvents are shown in Figure 1c. H_2_ mole fractions are determined from an average of three ^1^H NMR measurements with the sample degassed and repressurized between repeats, and the bars reflect the standard error.

**Table 1:** Henry’s Law Constants for H_2_ Solubility Measured for Solvents **1**–**16** Using ^1^H NMR Spectroscopy at 298 K^a^

solvent	inverse H_2_ Henry’s constant (MPa)	H_2_ Henry’s constant (mmol L^–1^ bar^–1^)	literature value (MPa)
deuterium oxide (**1**)	5094.7 ± 152.2	1.06 ± 0.03	7005.31^41^
methanol-*d*_4_ (**2**)	449.92 ± 17.26	5.6 ± 0.2	440.20 (314.4),^40^ 600–850^46^
acetonitrile-*d*_3_ (**3**)	394.54 ± 8.97	4.9 ± 0.2	545^42^
ethanol-*d*_6_ (**4**)	346.48 ± 7.04	4.9 ± 0.1	513^42^
dichloromethane-*d*_2_ (**5**)	367.91 ± 9.00	4.2 ± 0.1	537.63^43^
tetrahydrofuran-*d*_8_ (**6**)	226.51 ± 6.03	5.6 ± 0.2	370.02^44^
toluene (**7**)	190.18 ± 7.17	4.9 ± 0.2	330.92^45^
methylperfluorobutylether (**8**)	62.00 ± 1.64	10.0 ± 0.3	–
perfluorodecalin (**9**)	53.96 ± 1.78	7.6 ± 0.3	–
perfluorononane (**10**)	45.42 ± 1.49	8.8 ± 0.3	–
perfluorohexane (**11**)	22.15 ± 0.98	15.8 ± 0.7	–
perfluoro-1-decene (**12**)	20.14 ± 0.81	12.7 ± 0.5	–
methylperfluorocyclohexane (**13**)	19.36 ± 0.78	24.7 ± 1.0	–
perfluoroheptane (**14**)	18.96 ± 0.96	24.1 ± 1.2	–
1-bromoheptadecafluorooctane (**15**)	13.90 ± 0.49	28.9 ± 1.0	–
perfluorooctane (**16**)	8.28 ± 0.70	59.3 ± 5.0	–

a Values measured at 298 K; literature values are at the indicated temperature in brackets if different. Repeat measurements were performed by degassing the sample to remove H_2_, repressurizing, and rerecording ^1^H NMR spectra. Accordingly, values are quoted as an average of three measurements with a standard error.

Of all of the solvents examined, D_2_O exhibited the lowest H_2_ solubility, which confirms that H_2_ solubility will provide a limitation to performing highly efficient SABRE directly in aqueous solution. Notably, these data confirm that all the perfluorocarbons examined have significantly higher H_2_ solubility than the commonly used SABRE solvents methanol-*d*_4_, dichloromethane-*d*_2_, and chloroform-*d* and therefore they may be suitable for improved SABRE catalysis.

The extent to which the high H_2_ solubility of perfluorocarbons **8**–**16** can be exploited for SABRE hyperpolarization is examined. The study uses pyridine as a test substrate as it was one of the first molecules to be hyperpolarized using SABRE and it (and its derivatives) remains one of the most widely used and highest performing SABRE substrates.^12,14^ Accordingly, samples containing [IrCl(COD)(IMes)] (COD = *cis*-*cis*-1,5-cyclooctadiene and IMes = 1,3-bis(2,4,6-trimethylphenyl)-1,3-dihydro-2*H*-imidazol-2-ylidene) (2 mg) and pyridine (33 mM) in each of the solvents **8**–**16** (0.6 mL) were reacted with H_2_ (3 bar) for *ca* 2–3 h at 298 K. These samples were prepared as saturated solutions starting with 2 mg of iridium catalyst, with any undissolved catalyst removed from the solution prior to reaction with H_2_ by filtration through a syringe filter. After reaction, the H_2_ was replaced with *p*H_2_ and the samples were shaken for 10 s before being placed into the 9.4 T NMR spectrometer to record a hyperpolarized spectrum. The samples were shaken at 6.5 mT, 0.1 μT, or 0.4 μT for ^1^H, ^13^C, and ^15^N detection, respectively, as these are appropriate fields that yield efficient SABRE for the respective nucleus.^5,47−49^

When these measurements are performed, no observable hyperpolarization in solvents **9**, **10**, **11**, **14**, and **16** is achieved. Furthermore, only very weak ^1^H NMR signal enhancements (3-fold) are recorded for **13**. However, significant polarizations could be achieved for pyridine in perfluorocarbons **8**, **12**, and **15**. Of these, **8** performed the best with ^1^H, ^13^C, and ^15^N signal enhancements of 100-fold, 80-fold, and 25,000-fold, respectively, at 9.4 T (Table 2). In **15**, the ^1^H, ^13^C, and ^15^N NMR signal enhancements were roughly a factor of 2 lower than those in **8** (Table 2). Appreciable ^15^N polarization could also be achieved in **12**, although no enhanced ^13^C resonances were discerned and the associated ^1^H signal enhancements were less than 3-fold. In each case, the active SABRE catalyst is [IrCl(H)_2_(IMes)(pyridine)_2_] (see Section S3). This suggests that the nonpolar perfluorocarbons favor formation of neutral SABRE catalysts, departing from catalysts of the form [Ir(H)_2_(IMes)(pyridine)_3_]^+^ that form in polar organic solvents such as methanol.^50^

**Table 2:** NMR Signal Enhancements for Pyridine (33 mM)^a^

			no DMSO coligand			with DMSO coligand		
solvent	[IrCl(COD)(IMes)] solubility (mg mL^–1^)	maximum [IrCl(COD)(IMes)] (mM)	^1^H (fold)	^13^C (fold)	^15^N (fold)	^1^H (fold)	^13^C (fold)	^15^N (fold)
**8**	0.89	1.39	93 ± 6 (*ortho*)	77 ± 7 (*meta*)	25,413 ± 238 (8.3 ± 0.1%)	194 ± 10 (*ortho*)	55 ± 9 (*meta*)	2456 ± 27 (0.8 ± 0.1%)
			75 ± 6 (*meta*)			12 ± 8 (*meta*)		
			44 ± 3 (*para*)			188 ± 10 (*para*)		
**12**	0.22	0.34	3 ± 1 (*ortho*)	0	6,196 ± 131 (2.0 ± 0.1%)	7 ± 1 (*ortho*)	0	0
			2 ± 1 (*meta*)			4 ± 1 (*meta*)		
			1 ± 1 (*para*)			3 ± 1 (*para*)		
**15**	0.38	0.59	52 ± 1 (*ortho*)	0	14,750 ± 57 (4.8 ± 0.1%)	5 ± 1 (*ortho*)	0	0
			30 ± 2 (*meta*)			2 ± 1 (*meta*)		
			45 ± 2 (*para*)			3 ± 1 (*para*)		
**9**, **10**, **11**, **13**, **14**, **16**	<0.1	<0.14	0	0	0	0	0	0

a Starting with saturated solutions of [IrCl(COD)(IMes)] in perfluorinated solvents **8**–**16** at 9.4 T and 298 K after activation and exposure to *para*-hydrogen (3 bar) for 10 seconds with and without DMSO (25 mM). The shaking process was repeated 3 times and average signal enhancements are quoted with a standard error.

The observed ^1^H NMR signal enhancements for pyridine achieved in **8** and **12** could be improved slightly when DMSO was included as a coligand (25 mM). Specifically, the pyridine ^1^H NMR signal enhancements in **8** and **12** increased to 200-fold and 7-fold, respectively, although for **15** they dropped (Table 2). This change is reflected in the formation of neutral [IrCl(H)_2_(DMSO)(IMes)(pyridine)] as the SABRE catalyst (Figure 1a and Section S4). The pyridine dissociation rate from this catalyst in **8** was found using exchange spectroscopy (EXSY) to be 3.83 ± 0.02 s^–1^ at 298 K. This is close to the theoretically predicted optimal dissociation rate of 4.5 s^–1^ for related systems with a 1 Hz coupling between the hydride ligand and protons on the substrate.^51^ In these systems, a similar 1 Hz coupling is expected from theoretical calculations and experimental measurements on related systems.^48,52,53^ The much poorer ^1^H SABRE performance of [IrCl(H)_2_(IMes)(pyridine)_2_] is linked to its significantly faster pyridine dissociation rate, which was too rapid to measure at 298 K (1.66 ± 0.05 s^–1^ at 263 K). However, the resulting ^13^C and ^15^N NMR signal enhancements for [IrCl(H)_2_(DMSO)(IMes)(pyridine)] in **8** are *ca* 30% and 90% lower, respectively, than those achieved using [IrCl(H)_2_(IMes)(pyridine)_2_]. This confirms that faster substrate exchange rates are required to target ^15^N polarization using SABRE-SHEATH and is reflective of the larger ^1^H–^15^N *J* couplings associated with transfer to ^15^N.^52^

While pyridine seemed miscible with these solvents, when performing these hyperpolarization experiments, a significant challenge is associated with the low solubility of the [IrCl(COD)(IMes)] precatalyst. Therefore, we measured its solubility in **8**–**16** (Table 2) and found it to correlate strongly with the hyperpolarization response. For example, catalyst solubility in **9**–**11**, **14**, and **16** is less than 0.1 mg/mL and consequently SABRE efficiency is poor, despite high H_2_ solubility. This highlights the need for both high H_2_ and high catalyst solubility to enable efficient SABRE. Of solvents **8**, **12**, and **15**, the precatalyst solubilities follow the order **8** > **15** > **12**, which is also the order of their SABRE efficiency. At this point, solvents **9**–**16** were not explored further due to low catalyst solubility. It is worth noting that the unsaturated functionality of **12** can be hydrogenated by the SABRE catalyst. While this did not preclude pyridine hyperpolarization, hydrogenation of *ca* 10% **12** in the absence of pyridine over 24 h at room temperature was observed, which may further limit its utility as a solvent for SABRE catalysis.

Accordingly, the hyperpolarization of pyridine in **8** was repeated using dilute catalyst solutions to ensure that all the added catalyst dissolved, and hence any data are directly comparable. The amount of pyridine was also decreased to ensure a 1:20 ratio between catalyst and pyridine. Accordingly, [IrCl(COD)(IMes)] (1 mM) and pyridine (20 mM) were added to **8** (0.6 mL) and reacted with 3 bar of H_2_ for a few hours at room temperature. Now, shaking with *p*H_2_ at 298 K yields ^1^H NMR average signal enhancements for pyridine of 160 ± 10-fold (Figure 3a), which is between 2 and 4 times that found at the higher catalyst loadings. A greater improvement was seen for the ^15^N NMR signal enhancement, which increases from 8.3 ± 0.1% to 13.0 ± 1.1% (Figure 3b). The ^1^H NMR signal enhancements in **8** are similar to those achieved in **5** (dichloromethane-*d*_2_), but they can be up to an order of magnitude lower than in **2** (methanol-*d*_4_) when comparing samples with the same catalyst and pyridine concentrations (Figure 3a). However, the ^15^N polarization achieved in **8** (13.0 ± 1.1%) is similar to that in **5** (11.9 ± 1.5%) but significantly higher than that achieved under analogous conditions in **2** (8.5 ± 0.6%) (Figure 3b). We note that higher ^15^N enhancements can be achieved for pyridine in **2**(7,50) when more concentrated catalyst solutions are used, which cannot be employed for **8** due to low catalyst solubility in this solvent.

**Figure 3:**
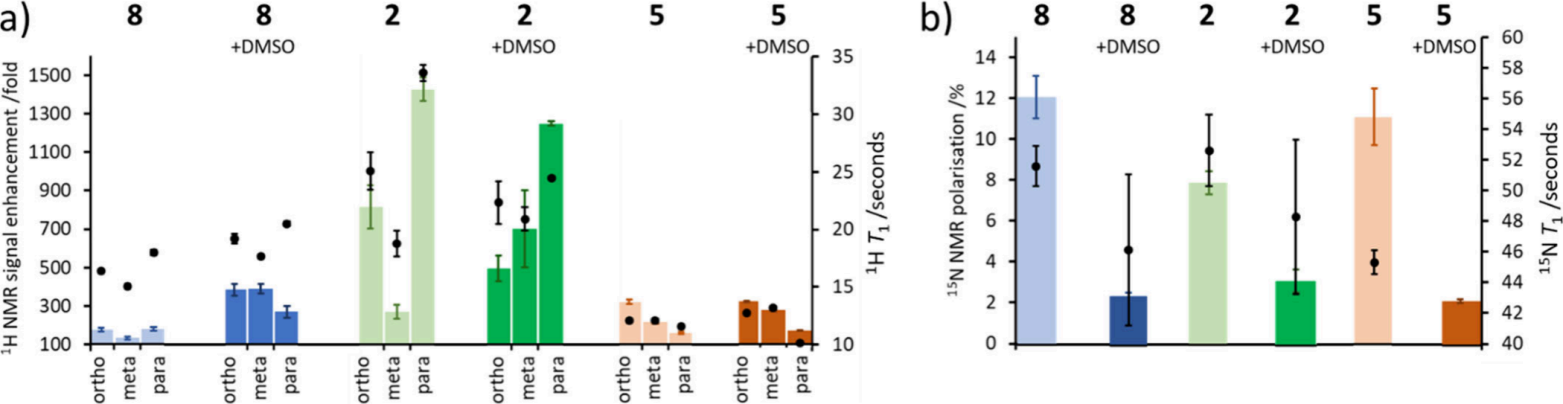
Attributed (a) ^1^H and (b) ^15^N SABRE-derived NMR signal enhancements for pyridine in the solvents **2**, **5**, and **8**, with and without addition of DMSO (5 mM) (bars, left-hand axis). Corresponding *T*_1_ values are shown (markers, right-hand axis). These data are recorded on samples that initially contained [IrCl(COD)(IMes)] (1 mM) and pyridine (20 mM) and 3 bar pH_2_ (0.6 mL). The signal enhancements result from shaking these samples for 15 s at (a) 6.5 mT and (b) 0.3 μT fields. The ^1^H and ^15^N NMR relaxation times were recorded using the hyperpolarized samples at 298 K and 9.4 T. Note that the ^15^N *T*_1_ in **5** with DMSO was too short to measure using hyperpolarized samples. The shaking process was repeated 3 times, and average signal enhancements are quoted with a standard error. For *T*_1_ times, standard errors were calculated by using a least mean squared approach to calculate the difference between the experimental data points and the fitted data for a single data set.

In order to rationalize these trends fully, the ^1^H and ^15^N *T*_1_ values for pyridine were measured for these samples at 298 K and 9.4 T (Figure 3). In addition, pyridine dissociation rates from the active catalyst were measured using EXSY. The pyridine ^1^H *T*_1_ values are longest in **2** and proved to be shortest in **5**. These values go some way to explain the higher ^1^H SABRE performance in **2**, but they do not completely reflect the trend in hyperpolarization efficiency as the factor of *ca* 2 shorter *T*_1_ in **5** compared to that in **8** does not account for the similar ^1^H SABRE efficiency in the two solvents. Similarly, *T*_1_ cannot solely explain the trends in ^15^N polarization (Figure 3). Hence, any difference in SABRE efficiency is likely to reflect additional factor(s), such as the pyridine dissociation rate from the different SABRE active species: neutral [IrCl(H)_2_(IMes)(pyridine)_2_] in **5** and **8** but charged [Ir(H)_2_(IMes)(pyridine)_3_]^+^ in **2**. Pyridine dissociation in **2**, the highest performing solvent for ^1^H SABRE, was 21.6 ± 1.6 s^–1^ at 298 K. Hyperpolarization efficiency decreases as this rate moves further from the theoretically predicted optimum (4.5 s^–1^).^51^ For example, in **5** the rate is faster at 135.9 ± 1.9 s^–1^ at 298 K and in **8** it is too fast to measure at 298 K (1.66 ± 0.05 s^–1^ at 263 K). For ^15^N SABRE-SHEATH, faster substrate exchange rate is preferred as a larger *J* coupling is involved in the spontaneous magnetization transfer at low field.^52^ Accordingly, the ^15^N polarization efficiency for the faster exchanging catalysts in **5** and **8** is higher compared to the more slowly exchanging **2**.

At dilute catalyst loadings, the effect of adding DMSO on the SABRE performance of pyridine in solvent **8** is similar to that at high catalyst loadings: ^1^H NMR signal enhancements increase whereas ^15^N signals decrease (Figure 3). These differences are linked to the slower exchange of DMSO-containing catalysts, as discussed previously, which improves ^1^H SABRE but reduces ^15^N SABRE-SHEATH efficiency. This is compounded by relaxation differences, as pyridine ^1^H *T*_1_ values extend slightly in **8** when DMSO is added (Figure 3), whereas those of ^15^N are shortened.

We finish by extending these measurements to a wider range of substrates including pyrazine, nicotine, and 3,5-dichloropyridine. Viable hyperpolarization of all of these substrates could be achieved in solvents **8**, **12**, and **15** (see Section S5), although **8** clearly proved superior. In the case of pyrazine and nicotine, the ^1^H and ^13^C NMR signal enhancements obtained in the perfluorocarbons were low (no higher than 200-fold), but significant ^15^N NMR signals at natural abundance could be achieved (3509 ± 166 or 1.1 ± 0.1% for pyrazine in **8** and 66,844 ± 612 or 21.8 ± 0.2% for nicotine, Figures 4a and 4b). Interestingly, for 3,5-dichloropyridine, ^15^N enhancements were now low, but the ^1^H NMR signal enhancements were significant (see Section S5). For example, the *ortho* and *para* protons of 3,5-dichloropyridine were enhanced by 1211 ± 25 and 455 ± 11-fold, respectively. This is comparable to the 1119 ± 14 and 870 ± 28 recently reported for this substrate in **2** at a much higher 5 mM catalyst loading.^48^ Such dihalogenated pyridines have already been reported to exhibit ^1^H relaxation times as long as 262 s^48^ in **5** and therefore they may not be shortened to the same extent in perfluorocarbon solvents as pyridine.

**Figure 4:**
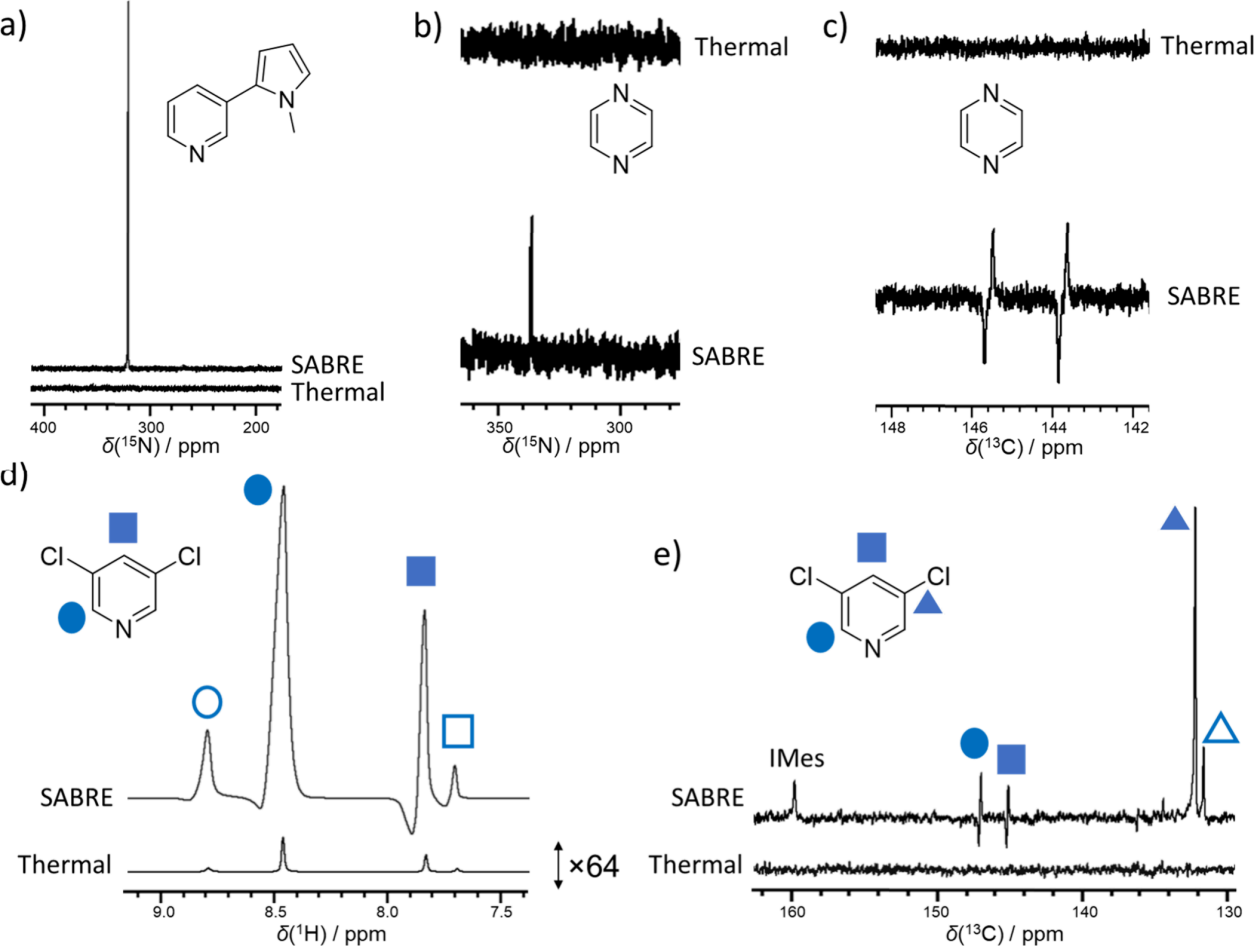
Representative hyperpolarized and thermally polarized NMR spectra for molecules hyperpolarized in perfluorocarbon solvents. (a) ^15^N NMR spectra for nicotine in **8**. (b) ^15^N and (c) ^13^C NMR spectra for pyrazine in **8**. Note that the downfield signal reflects free pyrazine whereas the upfield signal refers to pyrazine bound to the SABRE catalyst. (d) ^1^H and (e) ^13^C NMR spectra for 3,5-dichloropyridine in mixtures of **8** (80%) and **2** (20%). All spectra are at 9.4 T and 298 K, and all samples were prepared using [IrCl(COD)(IMes)] (saturated), substrate (25 mM), and H_2_ (3 bar) in 0.6 mL solvent. Parts (b)–(e) also contained DMSO (1.1 μL). Hyperpolarized spectra are recorded after shaking these samples for 10 s at (a) 0.2 μT, (b) 0.4 μT, (c) 0.1 μT, (d) 6.5 mT, and (e) 0.1 μT, respectively.

The beneficial effects of using mixtures of perfluorocarbons and organic solvents are also considered. The hyperpolarization of 3,5-dichloropyridine in a mixture of 80% **8** and 20% **2** was achieved at a 5 mM Ir loading as this could provide the combined benefits of higher H_2_ solubility than commonly used **2** and higher catalyst solubility than pure **8**. While ^15^N NMR signal enhancements were now significantly lower in this mixture than in **2**, ^1^H NMR signal enhancements of 2409 ± 292 and 911 ± 173 for the *ortho* and *para* sites, respectively, were recorded in this mixture and are *ca* 2 times higher than in either **2** or **8** (Figures 4d and 4e and Section S5).

In conclusion, this study establishes that perfluorocarbons (PFCs) **8**–**16** exhibit significantly higher hydrogen solubility, approximately 5–20 times greater, than the conventional solvents methanol, dichloromethane, and chloroform commonly used in SABRE catalysis. This high H_2_ solubility directly impacts the efficiency of hyperpolarization, enabling substrates like pyridine to achieve strong polarization transfer in PFCs such as methylperfluorobutylether (**8**), perfluoro-1-decene (**12**), and 1-bromoheptadecafluorooctane (**15**) where catalyst solubility using the standard [IrCl(COD)(IMes)] precatalyst is sufficient. Notably, methylperfluorobutylether emerged as the most promising solvent, facilitating enhancements of up to 1200-fold for ^1^H in 3,5-dichloropyridine and 25,000-fold (13.0% polarization) for ^15^N in pyridine (at natural abundance). These results are particularly significant, as they demonstrate that methylperfluorobutylether can outperform traditional solvents, such as methanol, in achieving SABRE polarization at comparable low catalyst loadings, highlighting its potential as an effective and biocompatible alternative. The study also suggests that using mixtures of PFCs and organic solvents could provide a route to increase SABRE efficiency further compared with using a conventional organic solvent alone.

The key challenge limiting SABRE efficiency in PFCs lies in the relatively low solubility of traditional SABRE catalysts within these solvents, which restricts the overall polarization efficiency despite enhanced H_2_ solubility. Addressing this limitation by better tuning of auxiliary ligands could be transformative for the application of PFCs in hyperpolarization processes. Future work could focus on developing perfluorinated catalysts^54,55^ specifically compatible with PFC solvents, thus removing this solubility barrier and unlocking higher SABRE efficiencies. Methods have already been developed to remove the iridium SABRE catalyst post hyperpolarization,^26,28,56,57^ which could be paired with PFC-based approaches to create biocompatible, hyperpolarized boluses. Such boluses, produced in low-toxicity PFCs, hold strong potential for *in vivo* applications, as they would provide a simple, safe, and efficient method to achieve high signal enhancements, expanding the utility of SABRE hyperpolarization in biomedical imaging and analytical studies.

## Supporting Information Available

Experimental methods, additional NMR spectra, and X-ray crystallography (PDF)
Transparent Peer Review report available (PDF)
