# Supplementary material for: High H2 Solubility of Perfluorocarbon Solvents and Their Use in Reversible Polarization Transfer from para-Hydrogen
Source: J Phys Chem Lett. 2025 Jan 6;16(2):510–7. doi: 10.1021/acs.jpclett.4c03190 (PMC11744794; doi:10.1021/acs.jpclett.4c03190)
Supplement: Supplementary file 1 — jz4c03190_si_001.pdf [file jz4c03190_si_001.pdf]

Supporting Information

High H_2 solubility of perfluorocarbon solvents and their use in reversible polarization transfer from *para*hydrogen

Callum A. Gater,[a,b] Orry J. Mayne,[a,b] Benjamin G. Collins,[a,b,c] Kieren J Evans,[a,b] Eleanor M. E. Storr,[a,b] Adrian C. Whitwood,[b] Daniel Watts,[c] Ben. J. Tickner,[a,b] and Simon B. Duckett[a,b]*

[a] Centre for Hyperpolarisation in Magnetic Resonance, University of York, Heslington, YO10 5NY, United Kingdom.

[b] Department of Chemistry, University of York, Heslington, YO10 5DD, United Kingdom.

[c] Department of Physics, Engineering and Technology, University of York, Heslington, YO10 5DD, United Kingdom.

*simon.duckett@york.ac.uk

Table of Contents

S1 Experimental

S2 Hydrogen solubility

S3 Formation of [Ir(Cl)(H)$_2$(IMes)(pyridine)$_2$] as the SABRE catalyst in perfluorocarbons

S4 Formation of [Ir(Cl)(H)$_2$(IMes)(pyridine)(DMSO)] as the SABRE catalyst in perfluorocarbons with DMSO coligand

S5 Hyperpolarization of a wider range of substrates in perfluorocarbons

S6 References

S1 Experimental

General

All NMR measurements were carried out on a 400 MHz Bruker Avance III spectrometer at 298 K unless otherwise stated. *Para*-hydrogen (pH_2) was produced by passing hydrogen gas over a spin-exchange catalyst (Fe_2O_3) at 28 K and used for all hyperpolarization experiments. This method produces constant p-H_2 with *ca* 99% purity. ^1H (400 MHz) and ^{13}C (100.6 MHz) NMR spectra were recorded with an internal deuterium lock. Chemical shifts are quoted as parts per million and referenced to residual solvent. All starting compounds were purchased from Sigma Aldrich, Fluorochem, or Alfa-Aesar and used as supplied without further purification. [IrCl(COD)(IMes)] was synthesised according to a literature procedure.[1] No isotopically labelled ^{13}C or ^{15}N compounds were used.

H_2 solubility

For H_2 solubility experiments, 0.7 mL of each solvent was prepared with an internal standard consisting of around 10 μL of dimethylsulfoxide or tetrahydrofuran depending on solubility in the solvent being probed. Volumes added were measured gravimetrically by mass difference using a Sartorius five decimal place balance to get an exact amount added. Each sample was degassed using five freeze thaw pump cycles to ensure removal of as much dissolved gas as possible from the solvent. Samples were then analysed using ^1H NMR spectroscopy at different H_2 pressures. Each sample was cooled whilst being pressurised using a dry ice acetone slurry to ensure that solvent loss was at a minimum throughout the experiment. When pressurising the sample was first filled and then shaken to equilibrate the gas dissolved before being filled and shaken again to ensure the pressure above the solvent was as quoted. This is to account for the fact that the pressure in the tube can drop from the set value when gas from the headspace dissolves in solution. The measurements were carried out in order of increasing pressure to avoid having to degas the solvent between each fill. The H_2 concentration was measured by integrating its resonance at *ca* 4.2 and scaling to account for the 25% fraction of undiscerned pH_2. The H_2 mole fraction was plotted as a function of H_2 pressure and Henry's constants extracted from the gradient of the line.

Hyperpolarization

The shake & drop method was employed for recording hyperpolarized SABRE NMR spectra. Samples were prepared in a 5 mm NMR tube that was fitted with a J. Young's tap. NMR samples were subsequently degassed by three freeze–pump–thaw cycles using a Schlenk line before filling the tube with H_2 or pH_2 at 3 bar overpressure. Once filled with pH_2, the tubes were shaken vigorously for 10 seconds at *ca* 6.5 mT for ^1H polarization, which is achieved in the stray field of our 9.4 T magnet. For ^{13}C and ^{15}N polarization, transfer fields of 0.1 μT and 0.4 μT are employed and are achieved using a mu metal shielded solenoid.[2] Immediately after that, the NMR tubes were put inside the spectrometer for immediate NMR detection. Both hyperpolarized and thermally polarized spectra were recorded on the same sample using the same spectrometer settings. NMR signal enhancements were calculated by dividing the hyperpolarized integral intensity by the corresponding intensity from a 1 scan thermal recorded and processed under the same conditions. Both hyperpolarized and thermally polarized spectra were recorded on the same sample using the same spectrometer settings. ^{13}C and ^{15}N NMR signal enhancements were calculated by reference to the thermally polarized solvent (for ^{13}C) or a standard solution of $^{15}NH_4Cl$ and are calculated according to equations previously reported. The shaking process was repeated 3 times and average signal enhancements are quoted with a standard error.

Exchange spectroscopy

The dissociation rate of pyridine was measured using exchange spectroscopy (EXSY). This involved the selective excitation of the *ortho* protons of bound pyridine B *trans* to hydride, followed by a variable delay time, before a ^1H NMR spectrum is recorded. Peaks are observed for excited metal complex, and as the delay time is increased a signal for free ligand that was previously bound to the metal centre becomes visible. The proportion of bound and free ligand as a function of delay time is measured and fitted to a two site kinetic model to extract a kinetic dissociation rate. The rate is found by minimising the difference between experimentally determined bound and free ratios, and those predicted by a kinetic two site exchange model. Errors were calculated using the Jack Knife approach. This involves sequentiually removing one data point, calculating the rate constant, and then taking an error of all of the rate constants.

T_1 Relaxation times

T_1 times were recorded on hyperpolarized samples by collecting a succession of 20 single scan ^1H or ^{15}N NMR spectra with a 12.6° pulse at 298 K that were separated by differing time intervals to capture the full signal decay profile. The decaying integral intensities were fitted to a model to extract a T_1 that has been reported previously.[3] T_1 times were calculated by fitting experimentally determined NMR integral intensities to values calculated using this model. Experimental integral intensities are normalised to one at their highest intensity in the first spectra. T_1 times were calculated using Microsoft Excel to give the smallest squared difference between experimental and modelled integral intensities. The standard errors quoted were calculated by using a least mean squared approach to calculate the difference between the experimental data points and the fitted data.

X-ray Diffraction

For X-Ray crystallography, suitable crystals were selected and mounted on an Oxford-Diffraction SuperNova dual-source X-ray diffractometer equipped with copper and molybdenum sources and a HyPix-6000HE detector. Cooling to 110 K was achieved using an Oxford Instruments Cryojet. A suitable crystal was selected and mounted on an Oxford-Diffraction SuperNova dual-source X-ray diffractometer equipped with copper and molybdenum sources and a HyPix-6000HE detector. Cooling to 110 K was achieved using an Oxford Instruments Cryojet. Using Olex2,[4] the structure was solved with the SHELXT[5] structure solution program using Intrinsic Phasing and refined with the SHELXL[6] refinement

package using Least Squares minimization. Details of the structural refinement and key parameters of the unit cell(s) are given in the appropriate sections.

S2 Hydrogen solubility

Table S1: Inverse Henry constants for H_2 dissolved in 1-16.

Solvent	Measured Inverse H_2 Henry's Constant			
	MPa	$mol^{-1}m^3bar$	$mol^{-1}m^3Pa$	$mol^{-1}m^3atm$
Deuterium oxide (**1**)	5094.7 ± 152.2	1.06 ± 0.03	94300 ± 2700	0.93 ± 0.03
Methanol-d_4 (**2**)	449.92 ± 17.26	5.60 ± 0.21	17900 ± 700	0.18 ± 0.01
Acetonitrile-d_3 (**3**)	394.54 ± 8.97	4.84 ± 0.19	20700 ± 800	0.20 ± 0.01
Ethanol-d_6 (**4**)	346.48 ± 7.04	4.90 ± 0.10	20400 ± 400	0.20 ± 0.01
Dichloromethane-d_2 (**5**)	367.91 ± 9.00	4.24 ± 0.10	23600 ± 600	0.23 ± 0.01
Tetrahydrofuran-d_8 (**6**)	226.51 ± 6.03	5.60 ± 0.15	17900 ± 500	0.18 ± 0.01
Toluene (**7**)	190.18 ± 7.17	4.91 ± 0.19	20400 ± 800	0.20 ± 0.01
Methylperfluorobutylether (**8**)	62.00 ± 1.64	9.96 ± 0.26	10000 ± 300	0.10 ± 0.01
Perfluorodecalin (**9**)	53.96 ± 1.78	7.55 ± 0.25	13200 ± 400	0.13 ± 0.01
Perfluorononane (**10**)	45.42 ± 1.49	8.80 ± 0.29	11400 ± 400	0.11 ± 0.01
Perfluorohexane (**11**)	22.15 ± 0.98	15.79 ± 0.70	6300 ± 300	0.063 ± 0.003
Perfluoro-1-decene (**12**)	20.14 ± 0.81	12.74 ± 0.51	7800 ± 300	0.077 ± 0.001
Methylperfluorocyclohexane (**13**)	19.36 ± 0.78	24.72 ± 1.00	4000 ± 200	0.040 ± 0.002
Perfluoroheptane (**14**)	18.96 ± 0.96	24.10 ± 1.22	4100 ± 200	0.041 ± 0.001
1-Bromoheptadecafluorooctane (**15**)	13.90 ± 0.49	28.92 ± 1.02	3400 ± 100	0.034 ± 0.001
Perfluorooctane (**16**)	8.28 ± 0.70	59.34 ± 5.02	1700 ± 100	0.002 ± 0.001

S3 Formation of [Ir(Cl)(H)$_2$(IMes)(pyridine)$_2$] as the SABRE catalyst in perfluorocarbons

Samples containing [IrCl(COD)(IMes)] (COD = *cis*-*cis*-1,5-cyclooctadiene and IMes = 1,3-bis(2,4,6-trimethyl-phenyl)imidazole-2-ylidene) (2 mg) and pyridine (1.6 μL, 33mM) in each of the solvents **8-16** (0.6 mL) were reacted with H_2 (3 bar) for *ca* 2-3 hours at 298 K. ^{1}H NMR spectra for the hydride regions collected using **8**, **12** and **15** are shown in Figure S1 as these solvents yielded pyridine hyperpolarization. In these cases, inequivalent hydride ligand signals were observed consistent with [Ir(Cl)(H)$_2$(IMes)(pyridine)$_2$]

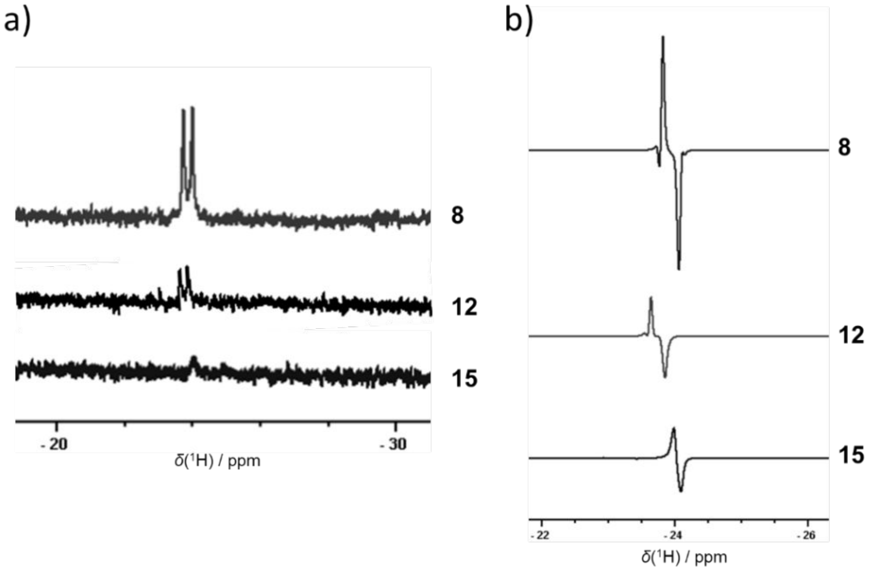

Figure S1: a) Partial multi-scan thermally polarized and b) single scan (90° pulse) hyperpolarized ^{1}H NMR spectra at 298 K and 9.4 T after [IrCl(COD)(IMes)] (2 mg) and pyridine (33 mM) are reacted with H_2 (3 bar) for ca 2-3 hours at 298 K in 8 (upper) 12 (middle) and 15 (lower). Spectra within each panel a) and b) are shown on the same vertical scale.

A solution containing [Ir(Cl)(H)$_2$(IMes)(pyridine)$_2$] in **8** was cooled to 245 K and characterised using 2D NMR. The structure of [Ir(Cl)(H)$_2$(IMes)(pyridine)$_2$] is shown in Figure S2 and its partial NMR resonances are given in Table S2. The complex was hard to fully characterise due to low catalyst solubility in **8** and large background signals for solvent **8** and free pyridine.

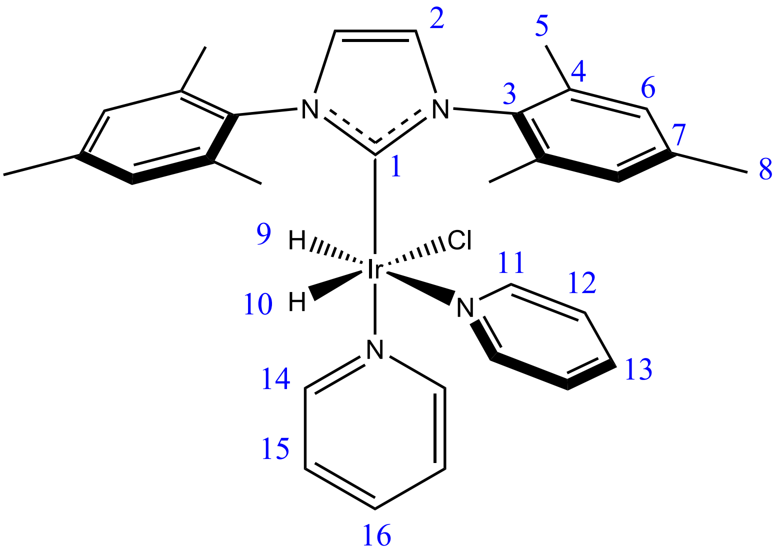

Figure S2: Structure of [Ir(Cl)(H)$_2$(IMes)(pyridine)$_2$], its NMR resonances are given in Table S2.

Table S2: Partial NMR resonances of [Ir(Cl)(H)$_2$(IMes)(pyridine)$_2$] in 8 at 245 K. The resonance labels correspond to those shown in Figure S2.

Resonance Number	^{1}H / ppm	^{13}C / ppm
1		-
2	-	122.37
3		-
4		134.02
5	2.28	-
6	6.74, 6.84	127.11
7		-
8	2.35	-
9	$-$ 23.48 / $-$ 23.82 (*d* $^2J_{HH}$ = 8 Hz)	
10	$-$ 23.48 / $-$ 23.82 (*d* $^2J_{HH}$ = 8 Hz)	
11	9.05	-
12	-	-
13	-	-
14	8.95	-
15	6.82	-
16	7.44	-

S4 Formation of [Ir(Cl)(H)$_2$(IMes)(pyridine)(DMSO)] as the SABRE catalyst in perfluorocarbons with DMSO coligand

Samples containing [IrCl(COD)(IMes)] (2 mg), pyridine (33 mM) and DMSO (25 mM) in each of the solvents **8-16** (0.6 mL) were reacted with H_2 (3 bar) for *ca* 2-3 hours at 298 K. ^{1}H NMR spectra for the hydride regions collected using **8**, **12** and **15** are shown in Figure S3 as these solvents yielded pyridine hyperpolarization. In these cases, inequivalent hydride ligand signals were observed with chemical shifts consistent with [Ir(Cl)(H)$_2$(IMes)(pyridine)(DMSO)].

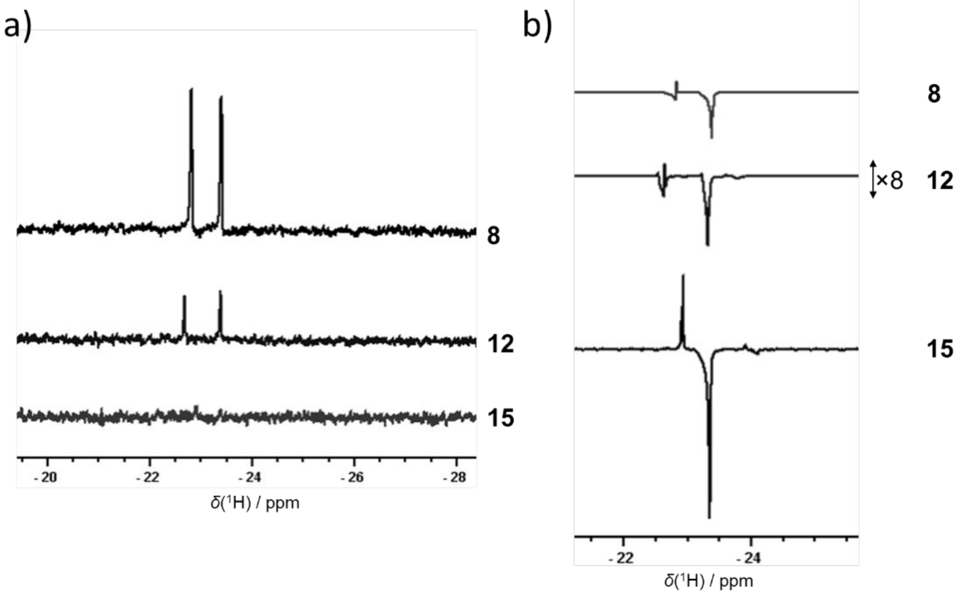

***Figure S3:** Partial multi-scan thermally polarized and b) single scan (90° pulse) hyperpolarized ^{1}H NMR spectra at 298 K and 9.4 T after [IrCl(COD)(IMes)] (2 mg), pyridine (33 mM), and DMSO (25 mM) are reacted with H_2 (3 bar) for ca 2-3 hours at 298 K in 8 (upper) 12 (middle) and 15 (lower). Spectra within each panel a) and b) are shown on the same vertical scale.*

A solution containing [Ir(Cl)$(H)_2$(DMSO)(IMes)(pyridine)] in **8** was cooled to 245 K and characterised using 2D NMR. The structure of Ir(Cl)$(H)_2$(DMSO)(IMes)(pyridine)] is shown in Figure S4 and its partial NMR resonances are given in Table S3. The complex was hard to fully characterise due to low catalyst solubility in **8** and large background signals for solvent **8** and free pyridine.

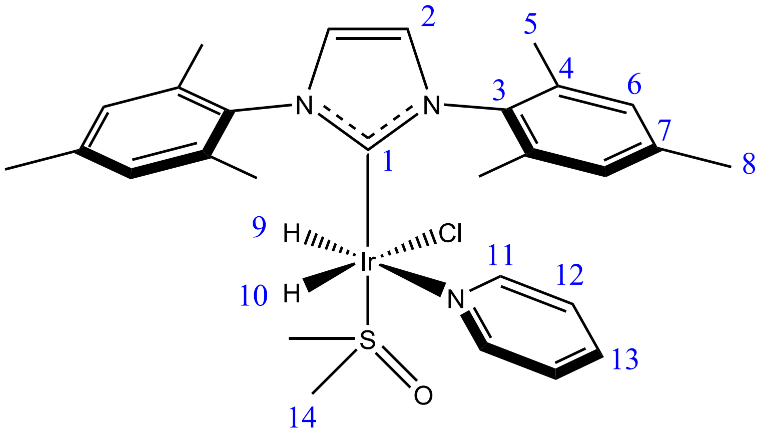

***Figure S4:** Structure of [Ir(Cl)$(H)_2$(DMSO)(IMes)(pyridine)], its NMR resonances are given in Table S3.*

***Table S3:** Partial NMR resonances of [Ir(Cl)$(H)_2$(DMSO)(IMes)(pyridine)]in 8 at 245 K. The resonance labels correspond to those shown in Figure S4.*

Resonance Number	^{1}H / ppm	^{13}C / ppm
1		-
2	6.63	121.60
3		-
4		135.51, 135.96
5	2.14, 2.12	-
6	6.73	128.11
7		138.33
8	2.18	-
9	− 22.82 / − 23.42 (*d*, $^{2}J_{HH}$ = 8 Hz)	
10	− 22.82 / − 23.42 (*d*, $^{2}J_{HH}$ = 8 Hz)	
11	8.86	-
12	6.93	-
13	7.39	-
14	2.87	-

After one of these samples in **8** was left for a week at room temperature, formation of single crystals were observed which were examined using X-ray crystallography. Some corresponded to the known [IrCl(COD)(IMes)] precatalyst, and other were found to be [Ir(Cl)$(H)_2$(IMes)(pyridine)(DMSO)]. Its crystal structure is shown in Figure S5 and its crystallographic details are given in Table S4.

Two different crystals for [Ir(Cl)$(H)_2$(IMes)(pyridine)(DMSO)] were observed: one was triclinic and the other trigonal. The triclinic crystal contained two molecules of iridium complex in the unit cell and exhibited considerable disorder. Within both complexes, the iridium, chloride and dimethylsufoxide were modelled in two positions in refined ratios of 0.9278:0.0722(8) and 0.8964:0.1036(12). The ADPs of disordered pairs of atoms were constrained to be equal (eg. Ir1 & Ir1a). Pairs of S-C bond were constrained to have the same length (S2-C56 & S2A-C56A, S1-C27 & S1A-C27A) as were the C-C distances C27-C28 & C27A-C28A. Hydrides were placed to align directly opposite the ligand *trans* to them with a Ir-H bond length of 1.7 angstroms. The DMSOs were also disordered and partially occupied with refined occupancies of 0.568(4):0.342(4) & 0.567(7):0.338(6). The ADPs of several pairs of atoms were constrained to be equal (C57 & C57A, C58 & C58A, C59 & C59A, C60 & C60A, S3 & S3A, O4 & O4A). C58 & C58A were constrained to occupy the same location as were C59 & C9A. There were two partially occupied solvent water molecules with refined occupancies of 0.5988 & 0.4797.

The trigonal crystal contained three [Ir(Cl)(H)2(IMes)(pyridine)(DMSO)] molecules and solvent which was too disordered to model with a discrete atom model. Therefore, a solvent mask was used which predicted two solvent zones per unit cell with volumes of 1036 and 198 cubic angstroms containing a predicted 252 and 48 electrons, respectively. There was evidence of disorder of two of the complexes with one in particular having large ADPs for the DMSO, chloride and pyridyl ligands as well as the iridium. This portion of the complex is surrounded by the disordered solvent which presumably explains the uncertainty of atom positions. Attempts to model using a two-position model were unsuccessful.

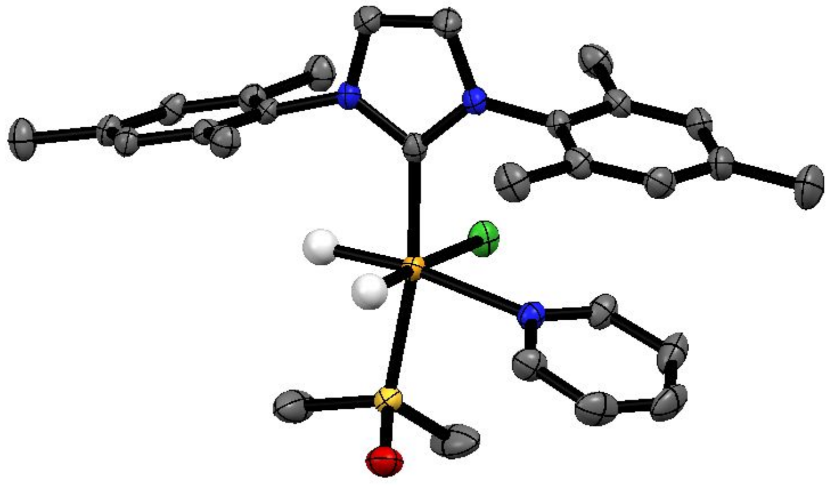

Figure S5: ORTEP plot for [Ir(Cl)(H)$_2$(IMes)(pyridine)(DMSO)]. Thermal elipsodids are shown at 50% probability and non-hydride hydrogen atoms and solvent of crystallisation etc. are omitted for clarity. Refinements details are given in Table S4.

Table S4: Crystal refinement details for [Ir(Cl)(H)$_2$(IMes)(pyridine)(DMSO)].

Empirical formula	$C_{29.82}H_{43.77}ClIrN_3O_{2.45}S_{1.91}$	$C_{28}H_{37}ClIrN_3OS$
Formula weight/ Da	772.19	691.31
Temperature/K	110.00(10)	110.00(10)
Crystal system	triclinic	trigonal
Space group	P-1	P-3
a/Å	13.6849(5)	30.0193(3)
b/Å	15.1620(7)	30.0193(3)
c/Å	17.0286(7)	17.3223(2)
α/°	81.800(4)	90
β/°	89.796(3)	90
γ/°	68.944(4)	120
Volume/Å^3	3259.3(2)	13518.8(3)
Z	4	18
ρ_{calc}g/cm^3	1.574	1.528
μ/mm^{-1}	10.075	10.249
F(000)	1551.0	6192.0
Crystal size/mm^3	0.1 × 0.073 × 0.033	0.21 × 0.14 × 0.05
Radiation	Cu Kα (λ = 1.54184)	Cu Kα (λ = 1.54184)
2Θ range for data collection/°	5.25 to 136.5	5.102 to 136.492
Index ranges	-16 ≤ h ≤ 14, -18 ≤ k ≤ 18, -20 ≤ l ≤ 20	-35 ≤ h ≤ 22, -28 ≤ k ≤ 35, -20 ≤ l ≤ 19
Reflections collected	43325	51374
Independent reflections	11957 [R_{int} = 0.0328, R_{sigma} = 0.0293]	16431 [R_{int} = 0.0310, R_{sigma} = 0.0321]
Data/restraints/parameters	11957/3/805	16431/0/976

Goodness-of-fit on F^2	1.203	1.048
Final R indexes [I>=2σ (I)]	R_1 = 0.0359, wR_2 = 0.0727	R_1 = 0.0365, wR_2 = 0.0885
Final R indexes [all data]	R_1 = 0.0376, wR_2 = 0.0734	R_1 = 0.0405, wR_2 = 0.0908
Largest diff. peak/hole / e Å^{-3}	1.49/-0.95	3.83/-1.66

S5 Hyperpolarization of a wider range of substrates in perfluorocarbons

Samples containing [IrCl(COD)(IMes)] (2 mg), substrate (5 equivalents relative to Ir) and in some cases DMSO (25 mM) in each of the solvents **8-16** (0.6 mL) were reacted with H_2 (3 bar) for *ca* 2-3 hours at 298 K. At this point the H_2 was replaced with pH_2 and the samples were shaken for 10 seconds before being placed into the 9.4 T NMR spectrometer to record a hyperpolarized spectrum. The samples were shaken either at 6.5 mT, 0.1 μT, or 0.4 μT for ^{1}H, ^{13}C or ^{15}N NMR spectra respectively.

S5.1 Pyrazine

Table S5: NMR signal enhancements when samples containing [IrCl(COD)(IMes)] (2 mg), pyrazine (5 equivalents relative to Ir) in the indicated solvents were hyperpolarized using SABRE, with or without coligand.

Solvent	No DMSO coligand			With DMSO-d_6 coligand		
	^{1}H /fold	^{13}C /fold	^{15}N /fold	^{1}H /fold	^{13}C /fold	^{15}N /fold
**8**	0	0	0	202 ± 9	148 ± 14	3509 ± 166 (1.1 ± 0.1%)
**12**	0	0	0	9 ± 1	0	0
**15**	3 ± 1	0	1527 ± 25 (0.5 ± 0.1%)	23 ± 1	0	0

S5.2 Nicotine

Table S6: NMR signal enhancements when samples containing [IrCl(COD)(IMes)] (2 mg), nicotine (5 equivalents relative to Ir) in the indicated solvents were hyperpolarized using SABRE. Note that $H_{o'}$, H_o, H_p and H_m denote the isolated ortho, ortho, para, and meta protons respectively. C_{ms} refers to the substituted meta carbon. The ^{15}N signal enhancement for 8 was recorded using 10 equivalents nicotine.

Solvent	NMR signal enhancement /fold		
	^{1}H /fold	^{13}C /fold	^{15}N /fold
**8**	$H_{o'}$: 56 ± 2<br>H_o: 38 ± 2<br>H_p: 38 ± 2<br>H_m: 25 ± 1	0	66,844 ± 612 (21.8 ± 0.2%)
**12**	$H_{o'}$: 34 ± 3<br>H_o: 30 ± 1<br>H_p: 25 ± 1<br>H_m: 12 ± 1	38 ± 6 (C_{ms})	10,317 ± 186 (3.4 ± 1.2%)
**15**	$H_{o'}$: 11 ± 1<br>H_o: 13 ± 2<br>H_p: 8 ± 1<br>H_m: 4 ± 1	62 ± 22 (C_{ms})	6,812 ± 310 (2.2 ± 0.1%)

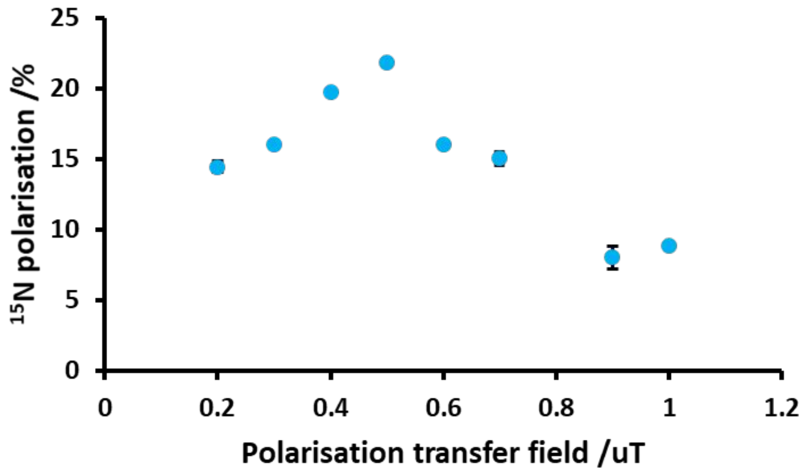

***Figure S6:** ^{15}N polarization for nicotine as a function of polarization transfer field. The sample used involved [IrCl(COD)(IMes)] (2 mg), pyridine (3.2 µL) reacted with H_2 (3 bar) for ca 2-3 hours at 298 K in **8**.*

S5.3 3,5-dichloropyridine

***Table S7:** NMR signal enhancements when samples containing [IrCl(COD)(IMes)] (2 mg), 3,5-dichloropyridine (5 equivalents relative to Ir), and DMSO (25 mM) in the indicated solvents were hyperpolarized using SABRE. Note that H_o and H_p denote the ortho and para protons respectively and C_o, C_m and C_p denote the ortho, meta and para carbon sites respectiveley.*

Solvent	NMR signal enhancement /fold		
	^{1}H /fold	^{13}C /fold	^{15}N /fold
**8**	H_o: 1211 ± 25<br>H_p: 455 ± 11	C_o: 95 ± 5<br>C_m: 130 ± 8<br>C_p: 94 ± 6	3221 ± 111<br>(1.1 ± 0.1 %)
**2**<br>(taken from ref no.[3]))	H_o: 1119 ± 14<br>H_p: 870 ± 28	C_m: 1221 ± 70	2606 ± 476
**8** (80%) **2** (20%)	H_o: 2409 ± 292<br>H_p: 911± 173	C_o: 116 ± 5<br>C_m: 671 ± 20<br>C_p: 96 ± 10	5234 ± 26<br>(1.7 ± 0.1 %)
**2**<br>(no DMSO coligand)<br>(taken from ref no.[3]))	H_o: 205 ± 4<br>H_p: 160 ± 3	C_o: 95 ± 5<br>C_m: 130 ± 8<br>C_p: 94 ± 6	0
**8** (80%) **2** (20%)<br>(no DMSO coligand)	H_o: 382 ± 57<br>H_p: 166 ± 34	0	0
**12**	H_o: 101 ± 9<br>H_p: 41 ± 2	C_m: 31 ± 2	14 ± 2
**15**	H_o: 24 ± 1<br>H_p: 10 ± 1	0	0

S6 References

1. Vazquez-Serrano, L. D., Owens, B. T. & Buriak, J. M. The search for new hydrogenation catalyst motifs based on N-heterocyclic carbene ligands. *Inorg. Chim. Acta* **359**, 2786–2797 (2006).
2. Iali, W. *et al.* Hyperpolarising pyruvate through signal amplification by reversible exchange (SABRE). *Angew. Chem.* **131**, 10377–10381 (2019).
3. Tickner, B. J. *et al.* Metal-Mediated Catalytic Polarization Transfer from para Hydrogen to 3, 5-Dihalogenated Pyridines. *ACS Cat.* **14**, 994–1004 (2024).
4. Dolomanov, O. V, Bourhis, L. J., Gildea, R. J., Howard, J. A. K. & Puschmann, H. OLEX2: a complete structure solution, refinement and analysis program. *J Appl Crystallogr* **42**, 339–341 (2009).
5. Sheldrick, G. M. SHELXT–Integrated space-group and crystal-structure determination. *Acta Crystallogr A Found Adv* **71**, 3–8 (2015).
6. Sheldrick, G. M. Crystal structure refinement with SHELXL. *Acta Crystallogr C Struct Chem* **71**, 3–8 (2015).
