# Supplementary material for: High H2 Solubility of Perfluorocarbon Solvents and Their Use in Reversible Polarization Transfer from para-Hydrogen
Source: J Phys Chem Lett. 2025 Jan 6;16(2):510–7. doi: 10.1021/acs.jpclett.4c03190 (PMC11744794; doi:10.1021/acs.jpclett.4c03190)
Supplement: Supplementary file 2 — jz4c03190_si_002.pdf [file jz4c03190_si_002.pdf]

jz-2024-03190m.R1

Name: Peer Review Information for "High H₂solubility of perfluorocarbon solvents and their use in re-versible polarisation transfer from parahydrogen"

First Round of Reviewer Comments

Reviewer: 1

Comments to the Author

The key challenge in applying parahydrogen-based hyperpolarization to biological in vivo imaging lies in reducing the toxicity of the chemical system while maintaining a high polarization level. This study employs perfluorinated solvents for SABRE hyperpolarization because the solubility of parahydrogen gas in perfluorinated solvents is higher compared to conventional deuterated solvents (e.g., water, methanol, chloroform, etc.). The authors investigated the solubility of hydrogen gas in a series of perfluorinated solvents and evaluated the effects on hyperpolarized signals of 1H, 13C, and 15N in these solvents. They studied the effects of catalyst solubility, T1 relaxation times, ligand exchange rates, and various substrates on the polarization enhancement factors in these solvents. This approach achieves high enhancement factors and polarization levels, and the conclusions are well-supported. This work shows potential for future application in developing ultra-sensitive magnetic resonance imaging agents via SABRE. I recommend acceptance of this manuscript after addressing the following points.

Comments:

1. The enhancement effect of SABRE hyperpolarization is directly related to the dissociation rate of the ligands, and significant changes in dissociation rates were observed after changing the solvent. Did the authors consider the influence of temperature on dissociation rates and enhancement factors in these perfluorinated solvents? Would it be possible to achieve an optimal dissociation rate, and thereby higher enhancement factors, through variable-temperature experiments?

2. The manuscript contains numerous instances where spaces are missing between words. For example, on page 1, left column, line 38: "limitationthat"; line 44, left column: "thatdetected"; and right column: "duringthe"; as well as in line 20 of the right column: "ItsJ." Similar issues are prevalent throughout the manuscript. The authors are advised to thoroughly check and correct these formatting errors.

3. In FIGURE 4, panels (b) and (c), the chemical shifts of the thermal polarization and SABRE NMR spectra are not aligned. Although the thermal spectrum does not show observable signals, these spectra should be overlapped within the NMR data processing software and exported as a single unified figure. Exporting spectra separately and later combining them into one figure is inappropriate. The boundaries of the figures are visibly misaligned in FIGURE 4.

4. On page 4, right column, line 20, the sentence “but a factor of between two and eleven times lower than in 2” contains an error. Please revise for clarity and correctness.

5. References 33 and 35 contain obvious errors. Please review and correct these entries.

Reviewer: 2

Comments to the Author

Gater et al. present their investigation into the use of the conventional IrIMes SABRE catalyst in fluorinated solvents. While the reported solubility values for [H_2] are informative, it is worth noting that some of these values likely were previously documented (see below). Additionally, it has been generally understood that gas solubility is significantly higher in fluorinated solvents than in organic solvents. Given the expertise of the authors, it could have been anticipated from the outset that this specific Ir-complex would exhibit limited solubility in these solvents, and suboptimal catalytic performance.

The manuscript would benefit from a more consistent approach to units, as the current variability complicates direct comparisons. Moreover, the description of the experimental data could be streamlined to enhance clarity, making it easier for readers to follow and appreciate the chemical findings and their broader implications.

There are numerous formatting issues, such as missing spaces between words, which detract from the readability of the manuscript. Notably, two prior works that focus on specially designed fluorinated Ir-catalysts are cited only briefly at the end of the manuscript. These references are directly relevant and should be integrated more thoroughly into the discussion, as they represent obvious candidates for comparison. Furthermore, no explicit comparisons are made to these studies, despite their significance. For example, the use of the hydrofluoroether Novec™ 7100 in Angewandte Chemie provides a relevant benchmark for the type of systems explored in this work. This work would be significantly improved when similar [H2] Henry’s constants and studies are repeated for the e.g. used Novec hydrofluroethers.

Questions / comments

- Abstract: "Future work could optimize catalyst solubility". This problem has not been introduced yet; therefore, looking for a solution seems a bit abrupt

- There are numerous text formatting issues (only first 3 are given):

- Introduction: "dynamics due to thehigh information" – check

- "polarisation, with the result thatdetected MR signals become" – check

- "significantly larger than those normallyrecorded.2 For- check"

- Figure 1: from the diagram, it is unclear what you filter in case of D2O. Why is the color white?

- How can one filter staff before redissolving in a methanol case? Should two steps be exchanged?

- Why is this structure used instead of two equatorially bound substrates? Comment that, in this case, NSub is pyridine.

- For each method in (b), the refs would be appropriate.

- "a fraction of the MR signal enhancement achievable in these organic solvents because of low pH2 solubility.26" – hm, given sufficient flow, the H2 concentration maybe not be an issue. There are works on the redissolution of IrIMes cat [https://pubs.acs.org/doi/10.1021/jp510825b 10.1021/acs.analchem.2c03682] and they report on the issues probably during the preparation process itself, for example, although activation is "irreversible". Other works [https://doi.org/10.1021/acs.jpcc.6b04484] do not have problems with the solubility of H2 in water but with the activity of the complex instead. So, I find that [H2] is too simple an explanation for such a complex effect where everything can affect final polarization, like maybe water coordination is competing with H2 or exchange rates are slower. These works are not acknowledged here.

- Table 1. Please define here used Henry's law and the constant. I know the law as c = H p, where H is the Henry's constant; The most practical units, in my opinion, would be mol/(L*bar) = M/bar. MPa are also ok but less practical as it does not provide direct comparison because solubility is multiplied by the molar mass and density ratio. Please write both numbers and definitions.

- "Of all the solvents examined, D2O exhibited the lowest H2 solubility, which is consistent with the reported low efficiency of SABRE in this solvent.26" – again, you want to pinpoint this to a simple fact, which is not the case. Different complexes are used in methanol and water (often), and there is a rich literature in tuning cats for methanol, but much fewer contributions on probing ligands for water-soluble cats.

- "Now that the high H2 solubility of perfluorocarbons 8-16" – would guess that you only check that. Please do some literature research. There are works where this fact is used (https://www.sciencedirect.com/science/article/pii/S2212982023000562) and works where it was measured (https://pubs.acs.org/doi/epdf/10.1021/je00008a012?ref=article_openPDF

- "samples were shaken at 6.5 mT, 0.1 μT, or 0.4 μT for 1H,4113C42 and 15N43detection" – references in supercrips are all mixed up

- "[IrCl(COD)(IMes)] (COD = cis-cis-1,5-cyclooctadiene and IMes = 1,3-bis(2,4,6-trimethylphenyl)-1,3-dihydro-2Himidazol-2-ylidene)(2 mg) and pyridine (1.6 μL) in each of the solvents 8-16 (0.6 mL) were reacted with H2 (3 bar) for ca 2-3 hours at 298 K." – Pls add one more table (or column to the table 1) with maximal achieved Ir concentrations? With NMR you can measure solubility of Ir in all of them. Please also write concentrations of substrates.

-"When these measurements are performed, low catalyst solubility prevents observable hyperpolarisation in solvents" – Maybe: We did not observe hyperpolarization in solvents 9…16; presumably…? – The data for [Ir] in various solvents is necessary to judge this claim. Later I found table 2, which was not cited here. You can still write [Ir] solubilities in different solvents for table 1 as these values are unknown. And again, mM are preferred units. Solubility in the presence of DMSO?

- The table 1 would also benefit from adding achieved hyperpolarization values that would support the claim of the solubility issues.

- "This change is reflected in the formation of neutral [Ir(Cl)(H)2(DMSO)(IMes)(pyridine)] as the SABRE catalyst," – do not see connection between increased SABRE in some solvents and decreased SABRE in other complexes and formation of this complex.

- “This confirmsthat faster substrate exchange rates are required to targetheteronuclear polarisation using SABRE-SHEATH,” – I would say faster exchange is compatible with hyperpolarization of heteronuclei because they have strong JNH, while not so strong JCH (https://analyticalsciencejournals.onlinelibrary.wiley.com/doi/full/10.1002/mrc.4694 https://pubs.acs.org/doi/10.1021/acs.jpclett.3c02980) hence it is not valid for all heteronuclei but with strong Js. Essentially, your observation confirms it.

- “This highlights the tension between high H2 and catalyst solubility.” – true for only one tested catalyst, right. The same is true in the above meantioned reference when they were trying to do exps in water.

-“The pyridine dissociation rate from this catalyst in 8was found using exchange spectroscopy (EXSY) to be 3.83 ± 0.02 s-1 at 298 K which is close to theoretically predicted optimal value of 4.5 s-1.45” – do you observe the solvent effect? Which exchange times do you have in methanol?

-Table 2. solubility now is in mgmL-1 but many things are in mM. for the clarity and convenience to compare things, please make all in mMs

-Table 2: “fold” units are in the headers, no need to write in the table.

-Figure 3: A-enhancement and b-polarizations. Let's use only polarization values.

Maybe you can color the T1 sale with red and all the corresponding symbols to it? I do not see T1 for 5+DMSO.

- You are shaking to dissolve H2. As a result of dissolution, the pressure goes down. Is it a significant effect, or do you think the pressure is still 3 bar after the shaking? I think “3 bar pH2 (0.6 mL)” is not so clear. For the shaking experiment, you should describe better sample volume, residual gas volume, estimated pressure, and corresponding calculated pressure after the shaking so one can estimate concentration. And in the same context as you do, shaking all these details is important for correctly estimating the pressure. Did you consider the pressure drop due to shaking? Is it significant in your settings?

- “This suggests that the nonpolar perfluorocarbons favour formation of neutral SABRE catalysts, departing from catalysts of the form [Ir(H)2(IMes)(pyridine)3]+ that form in polar organic solvents such as methanol.44” - interesting point, maybe you can change your figure 1 explaining / showing the difference of SABRE complexes in polar and nonpolar solvents?

-Figure 4. DMSO 1.1 uL, Talbe 2 DMSO 25 mM, Figure 3 DMSO-d6 5 mM. etc. please use unified units, mM. Why all different values? Was D6 always?

-Figure 4: “[IrCl(COD)(IMes)] (saturated)” – NMR gives you concentrations.

-Figure 4: there are some ugly horizontal and vertical lines around b, c, d and e panels

-Page 5:15-29: “Hence, any difference in SABRE efficiency is likely to reflect additional factor(s), such as the pyridine dissociation rate from the different SABRE active species: neutral [Ir(Cl)(H)2(IMes)(pyridine)2] in 5 and 8 but charged [Ir(H)2(IMes)(pyridine)3]+ in 2.Pyridine dissociation in 2, the highest performing solvent for 1H SABRE, was 21.6 ± 1.6 s-1 at 298 K. Hyperpolarisation efficiency decreases as this rate moves further from the theoretically predicted optimum (4.5 s-1).45 For example, in 5 the rate is faster at 135.9 ± 1.9 s-1 at 298 K and in 8 it is too fast to measure at 298 K (1.66 ± 0.05 s-1 at 263 K). For 15N SABRE-SHEATH, faster substrate exchange rate is preferred as a larger J coupling is involved in the spontaneous magnetisation transfer at low field. Accordingly, 15N polarisation efficiency for the faster exchanging catalysts in 5 and 8 is higher compared to the more slowly exchanging 2.

And

Page 3. 8-20 “The pyridine dissociation rate from this catalyst in 8was found using exchange spectroscopy (EXSY) to be 3.83 ± 0.02 s-1 at 298 K which is close to theoretically predicted optimal value

of 4.5 s-1.45 The much poorer 1H SABRE performance of

[Ir(Cl)(H)2(IMes)(pyridine)2] is linked to its significantly

faster pyridine dissociation rate, which was too rapid to

measure at 298 K (1.66 ± 0.05 s-1 at 263 K). However, the

resulting 13C and 15N NMR signal enhancements for

[Ir(Cl)(H)2(DMSO)(IMes)(pyridine)] in 8are ca 30% and 90%

lower respectively than achieved using

[Ir(Cl)(H)2(IMes)(pyridine)2]. This confirmsthat faster

substrate exchange rates are required to targetheteronuclear

polarisation using SABRE-SHEATH, and is reflective of the

different J couplings associated with transfer to these nuclei."

are very close in content sections.

-p5 LL17-18. Again defined SABRE.

-"dichloromethane commonly used in SABRE" what about chloroform? I thought that it was way more common.e.g. used in CASH-SABRE exp.

- "However, the key challenge limiting SABRE

efficiency in PFCs lies in the relatively low solubility of

traditional SABRE catalysts within these solvents, which

restricts the overall polarization efficiency despite the

enhanced H_2 solubility." – and untuned activity.

- "The study also suggests that using mixtures of PFCs and

organic solvents could provide a route to increase SABRE

efficiency further compared to using a conventional organic solvent alone." – but then you have to clean from them too!

- Figure 4. Did you use labeled compounds? Please indicate isotopic labeles, so one can see which are at n.a..

-“ theoretically predicted optimum (4.5 s-1).45” – is it indeed the prediction of the work? It is the estimate for the deltaJ of 1 Hz. Much larger J in case of 15N and much weaker in case of 13C (meantioned before too) and could be different for 1H. So, please revise your formulation.

Author's Response to Peer Review Comments:

Centre for Hyperpolarisation in Magnetic Resonance
DEPARTMENT OF CHEMISTRY
University of York, Heslington, YO10 5NY, United Kingdom

Corresponding Author:
Prof. S. B. Duckett
Direct Telephone + 44 (0)1904 322564
E-mail: simon.duckett@york.ac.uk

11th December 2024

Re: Revision requested for jz-2024-03190m

Dear Editor,

Please find attached a copy of a revised article entitled ‘**High H_2 solubility of perfluorocarbon solvents and their use in reversible polarisation transfer from *para*hydrogen’** which I hope satisfies all the reviewers comments and can move forward to publication in the Journal of Physical Chemistry Letters. We thank the reviewers for their detailed comments and provide a point by point response below.

Reviewer: 1

Recommendation: This paper is publishable subject to minor revisions noted. Further review is not needed. Comments: The key challenge in applying parahydrogen-based hyperpolarization to biological in vivo imaging lies in reducing the toxicity of the chemical system while maintaining a high polarization level. This study employs perfluorinated solvents for SABRE hyperpolarization because the solubility of parahydrogen gas in perfluorinated solvents is higher compared to conventional deuterated solvents (e.g., water, methanol, chloroform, etc.). The authors investigated the solubility of hydrogen gas in a series of perfluorinated solvents and evaluated the effects on hyperpolarized signals of 1H, 13C, and 15N in these solvents. They studied the effects of catalyst solubility, T1 relaxation times, ligand exchange rates, and various substrates on the polarization enhancement factors in these solvents. This approach achieves high enhancement factors and polarization levels, and the conclusions are well-supported. This work shows potential for future application in developing ultra-sensitive magnetic resonance imaging agents via SABRE.

I recommend acceptance of this manuscript after addressing the following points.

1. The enhancement effect of SABRE hyperpolarization is directly related to the dissociation rate of the ligands, and significant changes in dissociation rates were observed after changing the solvent. Did the authors consider the influence of temperature on dissociation rates and enhancement factors in these perfluorinated solvents? Would it be possible to achieve an optimal dissociation rate, and thereby higher enhancement factors, through variable-temperature experiments?

Temperature would certainly be a factor that would influence the exchange rate within the SABRE catalyst, and therefore the signal enhancement that can be achieved. One could in principle optimise the kinetics by variable temperature experiments. However, we decided to focus on room temperature experiments in this work due to a variety of factors. Primarily, we were concerned that changing the temperature would alter the catalyst and H_2 solubility and wanted to work under conditions where we knew the H_2 and Ir solubility in order to try and rationalise our experiments. We expected Ir solubility in the PFCs to drop at lower temperature, and in some solvents, such as DCM, much higher temperatures are precluded due to low solvent boiling points. For our current experimental setup (manual shaking) it is challenging to control precisely the temperature

during the pH_2 addition, shaking and transfer to magnet step. We will be sure to consider this suggestion in more detail as we seek to expand on this work in the future.

2. The manuscript contains numerous instances where spaces are missing between words. For example, on page 1, left column, line 38: "limitationthat"; line 44, left column: "thatdetected"; and right column: "duringthe"; as well as in line 20 of the right column: "ItsJ." Similar issues are prevalent throughout the manuscript. The authors are advised to thoroughly check and correct these formatting errors.

Thank-you for bringing these formatting errors to our attention. We think there must have been a problem with the conversion of our files during submission, as in our original word files there are appropriate spaces between these words. We will check our revised files for these errors.

3. In FIGURE 4, panels (b) and (c), the chemical shifts of the thermal polarization and SABRE NMR spectra are not aligned. Although the thermal spectrum does not show observable signals, these spectra should be overlapped within the NMR data processing software and exported as a single unified figure. Exporting spectra separately and later combining them into one figure is inappropriate. The boundaries of the figures are visibly misaligned in FIGURE 4.

We have now aligned the chemical shift axis of thermal and HP spectra for Figures 4b and c as suggested.

4. On page 4, right column, line 20, the sentence "but a factor of between two and eleven times lower than in 2" contains an error. Please revise for clarity and correctness.

We have amended this sentence and now say "These ^{1}H NMR signal enhancements in **8** are similar to those achieved in **5** (dichloromethane-*d*$_2$)**,** but can be up to an order of magnitude lower than in **2** (methanol-*d*$_4$), when comparing samples with the same catalyst and pyridine concentrations (Figure 3a).

5. References 33 and 35 contain obvious errors. Please review and correct these entries.

These references have been corrected.

Reviewer: 2

Recommendation: This paper may be publishable, but major revision is needed; I would like to be invited to review any future revision.

Comments: Gater et al. present their investigation into the use of the conventional IrIMes SABRE catalyst in fluorinated solvents. While the reported solubility values for [H_2] are informative, it is worth noting that some of these values likely were previously documented (see below). Additionally, it has been generally understood that gas solubility is significantly higher in fluorinated solvents than in organic solvents. Given the expertise of the authors, it could have been anticipated from the outset that this specific Ir-complex would exhibit limited solubility in these solvents, and suboptimal catalytic performance. The manuscript would benefit from a more consistent approach to units, as the current variability complicates direct comparisons. Moreover, the description of the experimental data could be streamlined to enhance clarity, making it easier for readers to follow and appreciate the chemical findings and their broader implications.

Even with our expertise, we were unable to predict the extent of solubility of [IrCl(COD)(IMes)] in each of these PFC's without measuring/studying it, which is what we have done here. We have shown that despite the limited solubility, catalytic performance can be high. Generally, we hope that the changes we have made throughout in response to the specific points below help to improve the readability and clarity of the work (see detailed responses below).

1. There are numerous formatting issues, such as missing spaces between words, which detract from the readability of the manuscript.

   Thank-you for bringing these formatting errors to our attention. We think there must have been a problem with the conversion of our files during submission, as in our original word files there are appropriate spaces between these words. Removal of these errors should significantly help to improve the readability and clarity of the work.

2. Notably, two prior works that focus on specially designed fluorinated Ir-catalysts are cited only briefly at the end of the manuscript. These references are directly relevant and should be integrated more thoroughly into the discussion, as they represent obvious

candidates for comparison. Furthermore, no explicit comparisons are made to these studies, despite their significance. For example, the use of the hydrofluoroether Novec™ 7100 in Angewandte Chemie provides a relevant benchmark for the type of systems explored in this work. This work would be significantly improved when similar [H2] Henry's constants and studies are repeated for the e.g. used Novec hydrofluroethers.

We agree that specially designed fluorinated Ir-catalysts in perfluorinated solvents are probably the way forward, which is why we have mentioned these works "Future work could focus on developing perfluorinated catalysts[46,47] specifically compatible with PFC solvents, thus removing this solubility barrier and unlocking higher SABRE efficiencies".

We are of the opinion that direct comparisons to the Angewandte Chemie work mentioned are not appropriate as that work primarily deals with achieving ^{13}C polarisation for pyruvate whereas our work focuses on ^{15}N and ^{1}H for N-heterocycles and the active SABRE catalyst is different. It is likely that the results (signal enhancements, exchange rates and solubility) will all be different for even minor changes to the IMes carbene ligand. We have chosen to focus on the commonly used and readily available IMes carbene, but a full study on the effect of introducing just a small number of F atoms to the carbene on solubility and SABRE would be interesting.

3. Abstract: "Future work could optimize catalyst solubility". This problem has not been introduced yet; therefore, looking for a solution seems a bit abrupt

Thank-you for bringing this to our attention. On reflection we agree and have removed his sentence from the abstract.

4. There are numerous text formatting issues (only first 3 are given): - Introduction: "dynamics due to thehigh information" – check - "polarisation, with the result thatdetected MR signals become" – check - "significantly larger than those normallyrecorded.2 For- check" Please see response to point 1.

5. Figure 1: from the diagram, it is unclear what you filter in case of D2O. Why is the color white?

In cases where hyperpolarisation is performed directly in D_2O, the water soluble Ir catalyst would still need to be filtered out (due to its toxicity). The caption of Figure 1 already states that "hyperpolarisation directly in aqueous solvent using a water-soluble SABRE catalyst which can be filtered out" The colour is supposed to be a very pale orange, but it does not look clear. We have amended Figure 1 to make this orange colour more obvious. This should help to reinforce visually that the purpose of the filtration is to remove the catalyst. We have changed "filter" in the diagram to "filter Ir".

6. How can one filter staff before redissolving in a methanol case? Should two steps be exchanged?

These steps are in the correct order. For example, the methanol solvent can be reduced by evaporation and at this point the catalyst precipitates. The solution is then filtered to remove the precipitated Ir and D2O is added to the agent in a small amount of methanol (eg https://onlinelibrary.wiley.com/doi/full/10.1002/anie.202306654). Experiments could be performed the other way round in which something is added to make an agent in methanol precipitate out. Filtration then collects the agent (and the catalyst stays dissolved in the methanol which is discarded). The filtered agent can then be redissolved in D2O (see https://pubs.acs.org/doi/full/10.1021/acssensors.2c01715). In principle, filtration could occur after redissolution, but these reports perform these steps in the order shown in Figure 1.

We have amended the Figure 1 caption to make this point clearer. We now say "These include (upper) hyperpolarisation in a methanolic solvent followed by sample concentration via solvent evaporation, filtration of the precipitated catalyst and redissolution to generate a catalyst-free aqueous bolus. Instead, the hyperpolarised agent could be precipitated and filtered out of the solution containing catalyst before being redissolved."

7. Why is this structure used instead of two equatorially bound substrates? Comment that, in this case, NSub is pyridine.

The structure in Figure 1 is used as this is the X-ray crystal structure of the active catalyst formed in this work (crystallographic details in the ESI). For perfluorocarbon solvents we

never form catalysts with two equatorially bound substrates. Instead we form either neutral [Ir(H)$_2$(Cl)(IMes)(pyridine)$_2$] or [Ir(H)$_2$(Cl)(DMSO)(IMes)(pyridine)] as detailed throughout the article.

The figure 1 caption has been amended to now include "..where NSub is an N-heterocycle e.g. pyridine."

8. For each method in (b), the refs would be appropriate. We have now included these in the caption of Figure 1b.

9. "a fraction of the MR signal enhancement achievable in these organic solvents because of low pH2 solubility.26" – hm, given sufficient flow, the H2 concentration maybe not be an issue. There are works on the redissolution of IrIMes cat [https://pubs.acs.org/doi/10.1021/jp510825b

10.1021/acs.analchem.2c03682] and they report on the issues probably during the preparation process itself, for example, although activation is "irreversible". Other works [https://doi.org/10.1021/acs.jpcc.6b04484] do not have problems with the solubility of H2 in water but with the activity of the complex instead. So, I find that [H2] is too simple an explanation for such a complex effect where everything can affect final polarization, like maybe water coordination is competing with H2 or exchange rates are slower. These works are not acknowledged here.

At constant pressure, the solubility of H_2 in a solvent will be a particular value, it should not be higher if H_2 is flowing over a solution (this would just replace used up oH_2 with pH_2) compared to a static environment. We note that both the mentioned works discuss the lower H_2 solubility in D_2O and discuss how it can be an obstacle to achieving high SABRE efficiency directly in D_2O. It was an oversight not to reference these two works and have now included them as citations. We have rephrased our statement and now say "….MR signal enhancement achievable in these organic solvents with SABRE efficiency likely hampered by lower *p*H_2 solubility."

We now say "Other approaches have used organic solvents for catalyst activation, before the active SABRE species is collected by solvent evaporation and redissolved in D2O where the

active Ir species is slightly more soluble than its precursor.[32] However, lower H_2 solubility in D_2O is still a factor that must be overcome."

10. Table 1. Please define here used Henry's law and the constant. I know the law as c = H p, where H is the Henry's constant; The most practical units, in my opinion, would be mol/(L*bar) = M/bar. MPa are also ok but less practical as it does not provide direct comparison because solubility is multiplied by the molar mass and density ratio. Please write both numbers and definitions.

We have now included an equation explicitly for Henry's law. We now say "Henry's law constants (H) (Table 1) were then calculated based on the linear relationship between gas vapour pressure (p) and measured concentration (c) according to Equation 1.[36–42] We have given inverse Henry's constants in units of MPa as most existing literature uses these units so it allows for comparison of our values to literature ones (see Table 1). We have also provided these values in other units in the supporting information (Table S1). We have now amended Table 1 to also include the Henry's constant values in values of M/bar as well.

11. "Of all the solvents examined, D2O exhibited the lowest H2 solubility, which is consistent with the reported low efficiency of SABRE in this solvent.26" – again, you want to pinpoint this to a simple fact, which is not the case. Different complexes are used in methanol and water (often), and there is a rich literature in tuning cats for methanol, but much fewer contributions on probing ligands for water-soluble cats.

We now say "Of all the solvents examined, D_2O exhibited the lowest H_2 solubility…..",

12. "Now that the high H2 solubility of perfluorocarbons 8-16" – would guess that you only check that.

Please do some literature research. There are works where this fact is used (https://www.sciencedirect.com/science/article/pii/S2212982023000562) and works where it was measured (https://pubs.acs.org/doi/epdf/10.1021/je00008a012?ref=article_openPDF

The first link discusses PFC no 9 (using our numbering). It does indeed suggest that the solvent is a good

H_2 carrier, but this is never quantified and no Henry constant is measured. The second link measures Henry's constants for two smaller PFC's (three carbons long) which is a different solvent to what we have used. We have now cited both and now say early on in the manuscript "Some perfluorocarbons are known to act as good H_2 carriers.[36,37]" We maintain there are no literature reported Henry's constants for 8-16 used in this work, although we would welcome being pointed to any references that we might have missed that have these values.

13. "samples were shaken at 6.5 mT, 0.1 µT, or 0.4 µT for 1H,4113C42 and 15N43detection" – references in supercrips are all mixed up

We have combined all the references at the end of this sentence.

14. "[IrCl(COD)(IMes)] (COD = cis-cis-1,5-cyclooctadiene and IMes = 1,3-bis(2,4,6-trimethylphenyl)1,3-dihydro-2Himidazol-2-ylidene)(2 mg) and pyridine (1.6 µL) in each of the solvents 8-16 (0.6 mL) were reacted with H2 (3 bar) for ca 2-3 hours at 298 K." – Pls add one more table (or column to the table 1) with maximal achieved Ir concentrations? With NMR you can measure solubility of Ir in all of them.

Please also write concentrations of substrates.

The maximum achieved Ir concentration could be calculated from the solubility. We have added these to the Table 2 for clarity. We have also replaced substrate volumes with concentrations.

15. "When these measurements are performed, low catalyst solubility prevents observable hyperpolarisation in solvents" – Maybe: We did not observe hyperpolarization in solvents 9…16; presumably…? – The data for [Ir] in various solvents is necessary to judge this claim. Later I found table 2, which was not cited here. You can still write [Ir] solubilities in different solvents for table 1 as these values are unknown. And again, mM are preferred units. Solubility in the presence of DMSO?

We now say "When these measurements are performed, no observable hyperpolarisation in solvents **9**,

10, **11**, **14** and **16**, and only very weak ^{1}H NMR signal enhancements (3-fold) are recorded for **13**." The Ir solubilities for the PFC solvents are given in Table 2 and we have now added maximum Ir concentrations in mM. The effect of DMSO on both the solubility of Ir and H2 is an interesting point. We have not measured if particular substrates (eg pyridine) or DMSO lead to changes in Ir solubility as these are present in small quantities in comparison to the substrate moles. The specific Ir solubility would depend on how much substrate and/or coligand (eg. other sulfoxides can be used) was there and might exhibit small differences for individual sample compositions. We intend our measured values to be used to help rationalise general trends in SABRE efficiency in these solvents.

16. - The table 1 would also benefit from adding achieved hyperpolarization values that would support the claim of the solubility issues.

These values are presented in Table 2

17. - "This change is reflected in the formation of neutral [Ir(Cl)(H)2(DMSO)(IMes)(pyridine)] as the SABRE catalyst," – do not see connection between increased SABRE in some solvents and decreased SABRE in other complexes and formation of this complex.

A significant part of the manuscript describes the effects that a different catalyst can have as different catalytic species will have very different pyridine dissociation rates. For example, we already say "This change is reflected in the formation of neutral [Ir(Cl)$(H)_2$(DMSO)(IMes)(pyridine)] as the SABRE catalyst, (Figure 1a and supporting information, section S4). The pyridine dissociation rate from this catalyst in **8** was found using exchange spectroscopy (EXSY) to be $3.83 \pm 0.02\ s^{-1}$ at 298 K which is close to theoretically predicted optimal value of $4.5\ s^{-1}$.[49] The much poorer ^{1}H SABRE performance of [Ir(Cl)$(H)_2$(IMes)$(pyridine)_2$] is linked to its significantly faster pyridine dissociation rate, which was too rapid to measure at 298 K ($1.66 \pm 0.05\ s^{-1}$ at 263 K)."

18. “This confirms that faster substrate exchange rates are required to target heteronuclear polarisation using SABRE-SHEATH,” – I would say faster exchange is compatible with hyperpolarization of heteronuclei because they have strong JNH, while not so strong JCH (https://analyticalsciencejournals.onlinelibrary.wiley.com/doi/full/10.1002/mrc.4694 https://pubs.acs.or g/doi/10.1021/acs.jpclett.3c02980) hence it is not valid for all heteronuclei but with strong Js.

Essentially, your observation confirms it.

We already say “For ^{15}N SABRE-SHEATH, faster substrate exchange rate is preferred as a larger *J* coupling is involved in the spontaneous magnetisation transfer at low field”. The mentioned statement has been amended to now say “This confirms that faster substrate exchange rates are required to target 15N polarisation using SABRE-SHEATH, and is reflective of the larger ^{1}H-^{15}N *J* couplings associated with transfer to 15N.[50]” An additional reference has been added as suggested.

19. “This highlights the tension between high H2 and catalyst solubility.” – true for only one tested catalyst, right. The same is true in the above meantioned reference when they were trying to do exps in water.

Noted

20. “The pyridine dissociation rate from this catalyst in 8 was found using exchange spectroscopy (EXSY) to be 3.83 ± 0.02 s-1 at 298 K which is close to theoretically predicted optimal value of 4.5 s-
1.45” – do you observe the solvent effect? Which exchange times do you have in methanol?

Yes, we have given these values and discussed how the pyridine exchange rate is different in different solvents (as in some cases the active catalyst is different) and generally, those solvents that give a rate closer to 4.5 s-1 give the best SABRE. We already say “Pyridine dissociation in **2**, the highest performing solvent for ^{1}H SABRE, was 21.6 ± 1.6 s^{-1} at 298 K. Hyperpolarisation efficiency decreases as this rate moves further from the theoretically predicted optimum (4.5 s^{-1}).[49] For example, in **5** the rate is faster at 135.9 ± 1.9 s^{-1} at 298 K and in **8** it is too fast to measure at 298 K (1.66 ± 0.05 s^{-1} at 263 K).

21. Table 2. solubility now is in mgmL-1 but many things are in mM. for the clarity and convenience to compare things, please make all in mMs

We have added maximum Ir concentrations to Table 2 in units of mM and retained solubility as a weight per volume unit.

22. Table 2: “fold” units are in the headers, no need to write in the table.

Extra “fold” has been removed from Table 2.

23. Figure 3: A-enhancement and b-polarizations. Let's use only polarization values.

We have retained enhancements as the units in Figure 3a. Our approach throughout is to use signal enhancements for ^{1}H and ^{13}C as these values are modest, and to quote % polarisation would give a small number that is not immediately easy to interpret intuitively. As ^{15}N enhancements are much more significant we give both the enhancement for consistency, but also the % polarisation which may be more intuitive for larger enhancements and allows easier comparison to other literature. Both enhancement and % polarisation are widely used in the literature.

24. Maybe you can color the T1 sale with red and all the corresponding symbols to it? I do not see T1 for

5+DMSO.

Red points for T1 could clash with existing colours on the figure, and might be hard for colour blind people to see. The figure 3 caption now contains a comment to clarify this point “Note that the ^{15}N T_1 in **5** with DMSO was too short to measure using hyperpolarised samples”.

25. You are shaking to dissolve H2. As a result of dissolution, the pressure goes down. Is it a significant effect, or do you think the pressure is still 3 bar after the shaking? I think “3 bar pH2 (0.6 mL)” is not so clear. For the shaking experiment, you should describe better sample volume, residual gas volume, estimated pressure, and corresponding calculated pressure after the shaking so one can estimate concentration. And in the same context as

you do, shaking all these details is important for correctly estimating the pressure. Did you consider the pressure drop due to shaking? Is it significant in your settings?

Upon shaking the H2 pressure does drop as the gas in the headspace goes into solution. It is for this reason that when H2 solubility measurements are performed the tube is pressurised to 3 bar, shaken, and this process repeated until the pressure remains at 3 bar (normally 2-3 cycles). In our experimental section (in the ESI) we already said "When pressurising the sample was first filled and then shaken to equilibrate the gas dissolved before being filled and shaken again to ensure the pressure above the solvent was as quoted" and have added "This is to account for the fact that the pressure in the tube can drop from the set value when gas from the headspace dissolves in solution" to clarify this point.

26. "This suggests that the nonpolar perfluorocarbons favour formation of neutral SABRE catalysts, departing from catalysts of the form [Ir(H)2(IMes)(pyridine)3]+ that form in polar organic solvents such as methanol.44" - interesting point, maybe you can change your figure 1 explaining / showing the difference of SABRE complexes in polar and nonpolar solvents?

After careful consideration, we have decided to retain the current Figure 1 as it contains an ORTEP Xray plot rather than a chemical structure. We do not have the comparable ORTEP plot for
[Ir(H)2(IMes)(pyridine)3]+ (it has been published elsewhere)

27. Figure 4. DMSO 1.1 uL, Talbe 2 DMSO 25 mM, Figure 3 DMSO-d6 5 mM. etc. please use unified units, mM. Why all different values? Was D6 always?

We have now replaced volumes with concentrations. The DMSO-d6 is a typo which has been corrected, we have used DMSO throughout.

28. Figure 4: "[IrCl(COD)(IMes)] (saturated)" – NMR gives you concentrations.

These concentrations are now included in Table 2.

29. Figure 4: there are some ugly horizontal and vertical lines around b, c, d and e panels

These should be omitted in our reformatted files

30. Page 5:15-29: "Hence, any difference in SABRE efficiency is likely to reflect additional factor(s), such as the pyridine dissociation rate from the different SABRE active species: neutral [Ir(Cl)(H)2(IMes)(pyridine)2] in 5 and 8 but charged [Ir(H)2(IMes)(pyridine)3]+ in 2. Pyridine dissociation in 2, the highest performing solvent for 1H SABRE, was 21.6 ± 1.6 s-1 at 298 K. Hyperpolarisation efficiency decreases as this rate moves further from the theoretically predicted optimum (4.5 s-1).45 For example, in 5 the rate is faster at 135.9 ± 1.9 s-1 at 298 K and in 8 it is too fast to measure at 298 K (1.66 ± 0.05 s-1 at 263 K). For 15N SABRE-SHEATH, faster substrate exchange rate is preferred as a larger J coupling is involved in the spontaneous magnetisation transfer at low field. Accordingly, 15N polarisation efficiency for the faster exchanging catalysts in 5 and 8 is higher compared to the more slowly exchanging 2.

And

Page 3. 8-20 "The pyridine dissociation rate from this catalyst in 8was found using exchange spectroscopy (EXSY) to be 3.83 ± 0.02 s-1 at 298 K which is close to theoretically predicted optimal value of 4.5 s-1.45 The much poorer 1H SABRE performance of [Ir(Cl)(H)2(IMes)(pyridine)2] is linked to its significantly faster pyridine dissociation rate, which was too rapid to measure at 298 K (1.66 ± 0.05 s-1 at 263 K).

However, the resulting 13C and 15N NMR signal enhancements for [Ir(Cl)(H)2(DMSO)(IMes)(pyridine)] in 8are ca 30% and 90% lower respectively than achieved using [Ir(Cl)(H)2(IMes)(pyridine)2]. This confirms that faster substrate exchange rates are required to target heteronuclear polarisation using SABRE-SHEATH, and is reflective of the different J couplings associated with transfer to these nuclei." are very close in content sections.

The first statements describe the solvent effect on dissociation rate and the second the effect of including

DMSO.

31. p5 LL17-18. Again defined SABRE.

This has been removed and only the acronym given

32. "dichloromethane commonly used in SABRE" what about chloroform? I thought that it was way more common. e.g. used in CASH-SABRE exp.

This has been amended to now say "than the conventional solvents methanol, dichloromethane and chloroform commonly used in SABRE catalysis"

33. "However, the key challenge limiting SABRE efficiency in PFCs lies in the relatively low solubility of traditional SABRE catalysts within these solvents, which restricts the overall polarization efficiency despite the enhanced H_2 solubility." – and untuned activity.

The subsequent sentence has now been amended to say "Addressing this limitation by better tuning of auxiliary ligands could be transformative for the application of PFCs in hyperpolarization processes."

34. "The study also suggests that using mixtures of PFCs and organic solvents could provide a route to increase SABRE efficiency further compared to using a conventional organic solvent alone." – but then you have to clean from them too!

We do not see this as an obstacle: If one is preparing a hyperpolarised contrast agent in an organic solvent they need to remove the solvent and catalyst for bioimaging. If higher signal enhancements are achieved in a mixture of miscible organic and PFC then one is in no worse position by still needing to do the same purification steps.

35. Figure 4. Did you use labeled compounds? Please indicate isotopic labeles, so one can see which are at n.a.

No ^{13}C or ^{15}N isotopically labelled compounds have been used in this work. We have added a statement in the experimental to highlight this.

36. " theoretically predicted optimum (4.5 s-1).45" – is it indeed the prediction of the work? It is the estimate for the deltaJ of 1 Hz. Much larger J in case of 15N and much weaker in

case of 13C (mentioned before too) and could be different for 1H. So, please revise your formulation.

The referenced work predicts an optimum exchange rate of 4.5 s^{-1} for a coupling of 1 Hz. We expect the J couplings within these types of active catalysts to be around 1 Hz and therefore an optimum rate not dissimilar to 4.5 s^{-1} can be expected. We now say "This is close to theoretically predicted optimal dissociation rate of 4.5 s^{-1} for related systems with a 1 Hz coupling between the hydride and substrate protons.[49] In these systems, a similar 1 Hz coupling is expected from theoretical calculations and experimental measurements on related systems.[46,50,51]"

A number of non-scientific changes have also been made to the manuscript including full author contact information and a complete reference no.17.

We hope you agree that we have sufficiently addressed the concerns of the reviewers and can proceed to publication.

Yours sincerely,

Prof. Simon Duckett

jz-2024-03190m.R2

Name: Peer Review Information for "High H₂solubility of perfluorocarbon solvents and their use in re-versible polarisation transfer from parahydrogen"

Second Round of Reviewer Comments

Reviewer: 2

Comments to the Author

Now, with better comprehensible numbers, I think the manuscript can be published. Please carefully revise the values in the tables, as now a solubility of 8 is lower than that of water. From my perspective, this table is the most helpful part together with table 2. Table 1 highlights the benefits of using fluorinated solvents. Table 2 highlights the need for a new catalyst. Therefore, the numbers there should be as correct as they can be. Because, in this case also, reproducibility is critical, it should be clearly described what we say as +- SE or STD and how it was evaluated, and how many replicates were done.

Abstract:

Hydrogen solubility in PFCs is shown to be an order of magnitude higher than in typical organic solvents. This high H2 solubility enables the PFCs to deliver substantial polarization transfer from parahydrogen, achieving up to 2,400-fold signal gains for 1H NMR detection and 67,000-fold (22% polarization) for 15N NMR detection at 9.4 T in substrates like pyridine and nicotine. Notably, methylperfluorobutylether outperforms catalytic efficiency in methanol-d4 and dichloromethane-d2 for pyridine at low catalyst loadings.

-I found some aspects of the abstract unclear and potentially challenging for readers to interpret. Specifically, it is difficult to discern whether certain points reflect established knowledge in the field or novel findings from this study.

“the low solubility of the [IrCl(COD)(IMes)] precatalyst. Therefore, we measured its solubility in 9-18 (Table 2), ” – probably 8 is correct here.

“This highlights the tension between high H2 and catalyst solubility.” – this is not a clear statement. I guess you want to say here, something like

This highlights the need for both high H_2 and catalyst solubility to enable efficient SABRE.

“At this point the solvents 12 and 15 were not explored further due to poorer catalyst solubility.” Not only 12 and 15, but all except for 8. – better is “At this stage, all fluorinated solvents 9-16 except for 8 were excluded from further consideration due to poor Ir catalyst solubility and suboptimal SABRE performance.”

“In order to rationalise these trends fully, the 1H and 15N T1 values for pyridine were measured for these samples (Figure 3). “ – at which field and temperature? Add here and in Figure 3 caption

“In order to rationalise these trends fully, the 1H and 15N T1 values for pyridine were measured for these samples (Figure 3). In addition, pyridine dissociation rates from the active catalyst were

measured using EXSY. " – I would split into two paragraphs for T1 and EXSY. Note that many lines in the EXSY description are identical to the text on the previous page.

You report, in many instances, values with standard deviation. Please comment on how it was evaluated. I would guess it was different for SABRE experiments and EXSY.

Figure 3. How many repetitions were done for each point? Do whiskers indicate standard deviation or error? How did you measure T1? How is it possible that you could not measure T1? What is the order of magnitude do you then expect?

Table 1 would look better in mmol, not so many zeros. Hm, 8 has h2 solubility of water? So, less than methanol. Is that correct?

Do you have an idea of the reason for the significant discrepancy between your measured solubility and the literature data?

Table 2 ")" is missing twice next to Imes if you like it in brackets.

Author's Response to Peer Review Comments:

Centre for Hyperpolarisation in Magnetic Resonance
DEPARTMENT OF CHEMISTRY
University of York, Heslington, YO10 5NY, United Kingdom

Corresponding Author:
Prof. S. B. Duckett
Direct Telephone + 44 (0)1904 322564
E-mail: simon.duckett@york.ac.uk

December 2024

Re: Revision requested for jz-2024-03190m

Dear Editor,

Please find attached a copy of a revised article entitled '**High H_2 solubility of perfluorocarbon solvents and their use in reversible polarisation transfer from *para*hydrogen'** which we hope can now proceed to publication in the Journal of Physical Chemistry Letters. We thank the reviewers for their comments and provide a point by point response below to the final changes they request.

Reviewer: 2

Recommendation: This paper is publishable subject to minor revisions noted. Further review is not needed.

Comments: Now, with better comprehensible numbers, I think the manuscript can be published.

1. Please carefully revise the values in the tables, as now a solubility of 8 is lower than that of water. From my perspective, this table is the most helpful part together with table 2. Table 1 highlights the benefits of using fluorinated solvents. Table 2 highlights the need for a new catalyst. Therefore, the numbers there should be as correct as they can be.

   We thank the reviewer for spotting the typo in the H_2 Henry's constant in 8, which should be 0.01, not 0.001 and is now corrected.

2. Because, in this case also, reproducibility is critical, it should be clearly described what we say as +- SE or STD and how it was evaluated, and how many replicates were done.

   Statements have now been added to the experimental to explain more clearly how these errors are calculated. We now say for signal enhancements "The shaking process was repeated 3 times and average signal enhancements are quoted with a standard error." and for T1's "The standard errors quoted were calculated by using a least mean squared approach to calculate the difference between the experimental data points and the fitted data." and for H2 solubility and "Repeat measurements were performed by degassing the sample to remove H_2 and repressurising as described and rerecording 1H NMR spectra. Accordingly, Henry's constants are quoted as an average of three measurements with a standard error. These statements are also included in the relevant figure captions and table headings.

3. Abstract: Hydrogen solubility in PFCs is shown to be an order of magnitude higher than in typical organic solvents. This high H2 solubility enables the PFCs to deliver substantial polarization transfer from parahydrogen, achieving up to 2,400-fold signal gains for 1H NMR detection and 67,000-fold (22% polarization) for 15N NMR detection at 9.4 T in substrates like pyridine and nicotine. Notably, methylperfluorobutylether outperforms catalytic efficiency in methanol-d4 and dichloromethane-d2 for pyridine at low catalyst loadings.

   I found some aspects of the abstract unclear and potentially challenging for readers to interpret. Specifically, it is difficult to discern whether certain points reflect established knowledge in the field or novel findings from this study.

   We have amended our abstract to now say "Hydrogen solubility in PFCs is shown here to be an order of magnitude higher than in typical organic solvents by determination of Henry's constants. We demonstrate how this high H_2 solubility enables the PFCs to deliver substantial polarization transfer from *para*hydrogen, achieving up to 2,400-fold signal gains for ^{1}H NMR detection and 67,000-fold (22% polarization) for ^{15}N NMR detection at 9.4 T in substrates like pyridine and nicotine."

4. "the low solubility of the [IrCl(COD)(IMes)] precatalyst. Therefore, we measured its solubility in 918 (Table 2), " – probably 8 is correct here.

   We have amended this to now say 8-16.

5. "This highlights the tension between high H2 and catalyst solubility." – this is not a clear statement. I guess you want to say here, something like

   This highlights the need for both high H_2 and catalyst solubility to enable efficient SABRE.

   This change has now been made

6. “At this point the solvents 12 and 15 were not explored further due to poorer catalyst solubility.” Not only 12 and 15, but all except for 8. – better is “At this stage, all fluorinated solvents 9-16 except for 8 were excluded from further consideration due to poor Ir catalyst solubility and suboptimal SABRE performance.”

   We now say “At this point the solvents **9-16** were not explored further due to low catalyst solubility”

7. “In order to rationalise these trends fully, the 1H and 15N T1 values for pyridine were measured for these samples (Figure 3). “ – at which field and temperature? Add here and in Figure 3 caption

   The temperature and magnetic field are now given in the text and the figure 3 caption

8. “In order to rationalise these trends fully, the 1H and 15N T1 values for pyridine were measured for these samples (Figure 3). In addition, pyridine dissociation rates from the active catalyst were measured using EXSY. ” – I would split into two paragraphs for T1 and EXSY. Note that many lines in the EXSY description are identical to the text on the previous page.

   We have commented on this point in our previous response and will retain our current descriptions

9. You report, in many instances, values with standard deviation. Please comment on how it was evaluated. I would guess it was different for SABRE experiments and EXSY.

   See answer to previous point no. 1

10. Figure 3. How many repetitions were done for each point? Do whiskers indicate standard deviation or error? How did you measure T1? How is it possible that you could not measure T1? What is the order of magnitude do you then expect?

    See answer to previous point no. 1. The explanation of how T_1 is given in the experimental (in the ESI). We have already commented in the previous revision that the

T1 for one sample was too short to measure using hyperpolarised ^{15}N NMR signals (if this signal is too low to detect in a single scan and you can't get more than at least 6-8 points you can't extract a T_1). In principle it could be measured using thermally polarised ^{15}N NMR, but this would take prohibitively long measurement times.

11. Table 1 would look better in mmol, not so many zeros. Hm, 8 has h2 solubility of water? So, less than methanol. Is that correct?

    The units were given in Table 1 in the format previously instructed by this reviewer. They have now been amended to mmol. Please see previous response to point no. 1

12. Do you have an idea of the reason for the significant discrepancy between your measured solubility and the literature data?

    There seems a large discrepancy generally within the literature for H_2 solubility values. For example, we can find literature values for H_2 solubility in methanol that vary wildly from 440 MPa in one reference to 600-850 MPa in other references that should be at comparable temperatures and pressures. We have added an additional reference that describes how measured H_2 solubility values can depend heavily on the experimental technique used to measure it.

13. Table 2 ")" is missing twice next to Imes if you like it in brackets.

    These typos have now been corrected

We hope that we can now proceed to publication.

Yours sincerely,

Prof. Simon Duckett
